# Antiretroviral Therapy at Conception Leads to Lower Peripheral CD49a^+^ NK Cells and Higher SERPINB2

**DOI:** 10.1155/jimr/4771787

**Published:** 2025-05-21

**Authors:** Yanling Huo, Jinhee Kim, Deborah Kacanek, Sadia Samer, Elizabeth G. Livingston, Elizabeth Stankiewicz Machado, Elena Martinelli

**Affiliations:** ^1^Center for Biostatistics in AIDS Research (CBAR), Harvard T.H. Chan School of Public Health, Boston, Massachusetts, USA; ^2^Department of Medicine Division of Infectious Diseases, Feinberg School of Medicine, Northwestern University, Chicago, Illinois, USA; ^3^Cell and Developmental Biology, Feinberg School of Medicine, Northwestern University, Chicago, Illinois, USA; ^4^Department of Obstetrics and Gynecology, Duke University School of Medicine, Durham, North Carolina, USA; ^5^Department of Infectious Disease, University Hospital and Instituto De Pediatria Martagão Gesteira (IPPMG)-Federal University of Rio De Janeiro, Rio De Janeiro, Brazil

## Abstract

**Problem:** Antiretroviral therapy (ART) during pregnancy is essential to prevent vertical HIV transmission and preserve the health of the mother and child. However, ART in pregnancy has been associated with adverse birth outcomes linked to poor placental development. Immune dysregulation of placental development is an important factor in the development of preeclampsia (PE), a common hypertension disorder of pregnancy. Some studies found an association between ART use at conception or during the first trimester and PE. However, little is known regarding the impact of timing of ART initiation on the immune environment in pregnancy.

**Methods:** To investigate the immune environment in pregnant persons with HIV (PPWH) on ART at conception (*N* = 40) compared to PPWH that started ART in the second trimester (*N* = 40) we analyzed specimens from the International Maternal Pediatric Adolescent AIDS Clinical Trials (IMPAACT) Perinatal Core Protocol, P1025, concluded in 2013.

**Results:** No difference was found in soluble factors in circulation including PlGF and sFLt-1, associated with PE. However, upon analysis of PBMC by high dimension flow cytometry, we detected a lower frequency of circulating CD49a^+^ NK cells, associated with uterine tissue and pregnancy, in PPWH on ART at conception compared with PPWH who started ART in the second trimester. Moreover, PBMC from PPWH on ART at conception expressed higher levels of SERPINB2 in transcriptomics analyses.

**Conclusions:** Our findings shed new insights into the potential impact of ART at conception and suggest the persistence of a dysregulated inflammatory environment compared to PPWH starting ART after the conclusion of placental development.

## 1. Introduction

Antiretroviral therapy (ART) during pregnancy is critical to preserve maternal health and prevent vertical transmission of HIV. Every year, over 1 million persons living with HIV (PLWH) on ART become pregnant [1]. Hence, a better understanding of the impact of HIV and ART in pregnancy is essential to improve maternal and newborn health. Until 2013 [2], sameday ART initiation for persons newly diagnosed with HIV was not mandatory in pregnancy and in most cases, providers waited until the second trimester to initiate therapy. In contrast, today, most PLWH are already on ART at the time of conception or sameday ART initiation occurs independent of gestational age. However, studies focused on understanding the impact and safety of ART use at conception or initiation of antiretrovirals (ARVs) during the first trimester compared to initiation of ART after the first trimester are limited [1].

Moreover, higher rates of preterm birth and low birthweight are associated with ART utilization in pregnancy and, in particular, with protease inhibitors (PI)-based ART [3, 4]. This has been linked to a PI-mediated decrease in progesterone levels during pregnancy [5]. However, placental abnormalities including hypervascularity, increased maternal vascular malperfusion (MVM), and decreased placental weight are all associated with ART-use in pregnancy [6]. This appears to be independent of the specific ARV regimen, but it may be associated with timing of ART initiation before or soon after conception [7].

Hence, the effect may not be limited to PI-based ART, but it may be rather linked to the effect of a combination of HIV and ART (any) on placental development.

Impaired placental development and function are, in turn, the most critical determinants of preterm birth and low birthweight [8]. However, they also contribute to the development of preeclampsia (PE) and other hypertensive disorders of pregnancy (HDP) [9]. HDP are a leading cause of maternal mortality as well as long-term morbidity, doubling the risk for cardiovascular disease later in life [10]. Moreover, HDP are key drivers of low birthweight, linking PE with other disorders associated with HIV and ART. Studies from the early ART era (pre-integrase inhibitors) suggested that pregnant people with HIV (PPWH) on ART had higher risk of HDP and particularly PE than PPWH who were ART naïve [11–13]. Other studies did not find this association [14, 15, 16]. Systematic reviews also indicate contradicting results adding to the confusion and highlighting the need of larger and better controlled studies. Of note, most of the studies of associations between ART and PE did not distinguish ART at conception or ART initiated during the First trimester from ART initiated during the second or third trimester of pregnancy [17–19]. Importantly, ART use at conception was associated with higher risk of PE and low birthweight in several studies that made this distinction [15, 20, 21]. On the other hand, a recent large retrospective study conducted in South Africa, did not find an association between ART at conception and HDP of pregnancy in pregnancies occurring after 2016 when the universal ART era started [14–16, 22].

With sameday ART initiation for new infections in pregnancy, a substantial number of women initiate ART during the first trimester, leaving open the possibility that ART initiation around the time of conception or during the first trimester increases the risk of adverse pregnancy outcomes, particularly those linked to impaired placental development.

PE is a heterogeneous syndrome and the result of placental dysfunction [23]. Although its precise etiology is unclear, the disease process is hypothesized to begin at implantation with an immune-mediated dysregulation of placentation [24]. Immune imbalances during implantation result in poor trophoblast invasion which leads to poor spiral artery remodeling and insufficient placental perfusion with resultant placental ischemia or hypoxia [25–27].

We sought to identify possible immune-mediated mechanisms associated with systemic effects of ART at conception compared to ART initiation in the second trimester, using data collected from the International Maternal Pediatric Adolescent AIDS Clinical Trials (IMPAACT) P1025 study Perinatal Core Protocol. The study was a large cohort study (~3000 women), designed to assess safety and effectiveness of mother-to-child transmission interventions that enrolled women living with HIV from 2008 to 2013 [28–32], prior to the universal ART era. Nearly half of the women in the study started ART in the second trimester. This allowed us to compare inflammatory and immune biomarkers linked to placental disfunction and dysregulated placental development in women on ART at conception (*n* = 40) against women that started therapy after the conclusion of placentation (gestational week [GW] ~ 13; *n* = 40). Although sample collection and storage were highly heterogenous, we found that women who were on therapy at conception had lower frequencies of CD49a-expressing NK cells and higher levels of SERPINB2 expression in PBMC.

## 2. Materials and Methods

### 2.1. Study Population and Inclusion/Exclusion Criteria

The IMPAACT P1025 study was a multicenter prospective observational study in the US that enrolled women living with HIV and their infants from 2002 to 2013 and was designed to assess safety and effectiveness of mother-to-child transmission interventions [28–32]. P1025 study was approved by institutional review boards at each participating site and written informed consent was obtained from women enrolled in study. Eligible for inclusion in this analysis were women enrolled in P1025, whose first pregnancy (primigravida) was a live birth singleton and had at least three vials of >150 μL/each plasma collected between 20 and 36 weeks of gestation and received an ART regimen with at least three drugs (ART) in pregnancy. Eligible women were further classified into two groups based on timing of ART initiation with further inclusion criteria: (1) the on ART at conception group, which included women on ART at the time of conception or initiated ART ≤ 3 weeks gestation and with no drug interruption any time during the first trimester and within 6 weeks before sample collection; (2) the ART initiated in the second trimester group, which included all women who initiated ART after GW 13 and no drug interruption within 6 weeks before sample collection. For both groups, a single drug switch and a period on only one drug >6 weeks before sample collection were allowed. The two groups of women were matched on the gestational age at specimen collection (categorized as 20–23 versus 24–27 versus 28–31 versus 32 versus 33 versus 34 versus 35 versus 36 weeks) with a 1:1 ratio.

Exclusion criteria included diagnosis (at any time during pregnancy) of a chronic, genetic, and untreatable conditions with a systemic impact on the immune system (including autoimmune diseases, cancer, and tuberculosis [TB]) and a diagnosis, concomitant with sample collection, of systemic infections or nonsystemic infection with a known systemic inflammatory component.

Finally, women with a diagnosis of PE predisposing factors (including chronic and pregnancy related hypertension, diabetes, PE and hemolysis, elevated liver enzymes and low platelets [HELLP] syndrome) were included. This is because these conditions occur independent of ART timing and, hence, are likely equally present in the two groups.

### 2.2. Inflammatory and Soluble Factors Detection in Plasma

We used a combination of customs Luminex and MSD plex assays and a TGF-*β* enzyme-linked immunosorbent assay (ELISA) (Thermo Fisher) to measure soluble factors in the plasma of the 80 PPWH. Plasma was diluted 1:2 in all assays and the assay followed manufacturer instructions. The custom assays included analysis of 19 analytes by Luminex (CCL3, SDF-1*α*, IL-1*β*, IL-2, CXCL10, IL-6, Eotaxin, IL-13, IL-1RA, CCL5, TNF-*α*, CCL4, CCL2, P-Selectin, IL-15, IL-18, ICAM-1, E-Selectin, VEGF-A), 10 by MSD U-Plex (Ang-1, Ang-2, GM-CSF, IFN-*γ*, IL-10, IL-12p70, IL-17A, IL-4, IL-5, and PlGF) and an MSD V-Plex for sFLt-1. A set of 20 samples from a different, pilot study was used for validation of these custom platforms.

### 2.3. PBMC Phenotype by Flow Cytometry

PBMC from each of the 80 PPWH were thawed and divided into four parts for staining with four panels of antibodies for the analysis of T cell subsets, Tregs, NK cells, and dendritic cells (DCs). The full list of antibodies in each panel can be found in Supporting Information Table S5. Live/dead Aqua (Thermo Fisher) was used for viability.

The data were analyzed with FlowJo vers 10.8 by classical gating of population of interest and, separately, also with a high dimension analysis pipeline. Specifically, clustering analysis was performed with FlowSOM [33] with eight populations (six and ten populations were tested as well without difference in the results) and each population was directly compared between the two groups.

### 2.4. Bulk RNAseq of PBMC

Total RNA was extracted from a subset of 20 PPWH per group (chosen based on sample availability and to maintain matched characteristics) using the RNAeasy Kit (Qiagen) following manufacturer recommendations. Due to the low RNA yield, a low input RNA-seq library preparation was performed, and samples were sequenced at the NUSeq Core using three lanes of the Illumina HiSeq 4000 (50 bp, single-read) yielding >22 M reads per sample. Data were analyzed using the Partek Flow software. Sequencing data were demultiplexed, trimmed, and aligned to hg38 with STAR 2.7.8a. Differential gene expression analysis using the quantified gene transcripts was performed with DESeq2 R package [34] comparing the samples in the two groups. A cutoff of false discovery rate (FDR) <0.1 was used for significance. Differentially expressed genes (DEGs) were analyzed by gene set enrichment analysis (GSEA) to identify enriched pathways and molecular processes in the ART at conception group compared to the second trimester group.

### 2.5. Statistical Analysis

Characteristics of the analysis population were summarized by ARV exposure group (on ARV at conception, initiated at ≥second trimester). The concentrations of inflammatory biomarkers were log_10_-transformed to approximate a normal distribution, and mean log_10_ concentration levels were compared between the two groups of women using a two sample *t*-test. Mean and median of the % flow immune biomarkers as well as each population obtained with FlowSOM were summarized and compared between the two groups using the Wilcoxon rank-sum test. For women whose inflammatory biomarker concentration was below the lower limit of detection (LLOD) and therefore was not obtained, the value of LLOD was used in the analysis. For concentration levels that were obtained but were below the lower limit of quantification (LLOQ) or above the upper limit of quantification (ULOQ), the actual reported extrapolated concentrations were used in the analysis. Principal component analysis (PCA) was conducted for the log-transformed inflammatory biomarkers to identify cluster patterns based on the retained component scores. The analysis did not account for multiple comparisons that would inflate the type I error, as this was a descriptive type of analysis. A subset analysis was performed excluding samples from PPWH that were viremic (>400 copies/mL) or had low CD4 counts (<350 cells/uL). SAS version 9.4 was used for statistical analysis of flow cytometry and soluble factors data and partek Flow was used for RNAseq data analysis.

## 3. Results

### 3.1. Study Population

**Figure 1:**
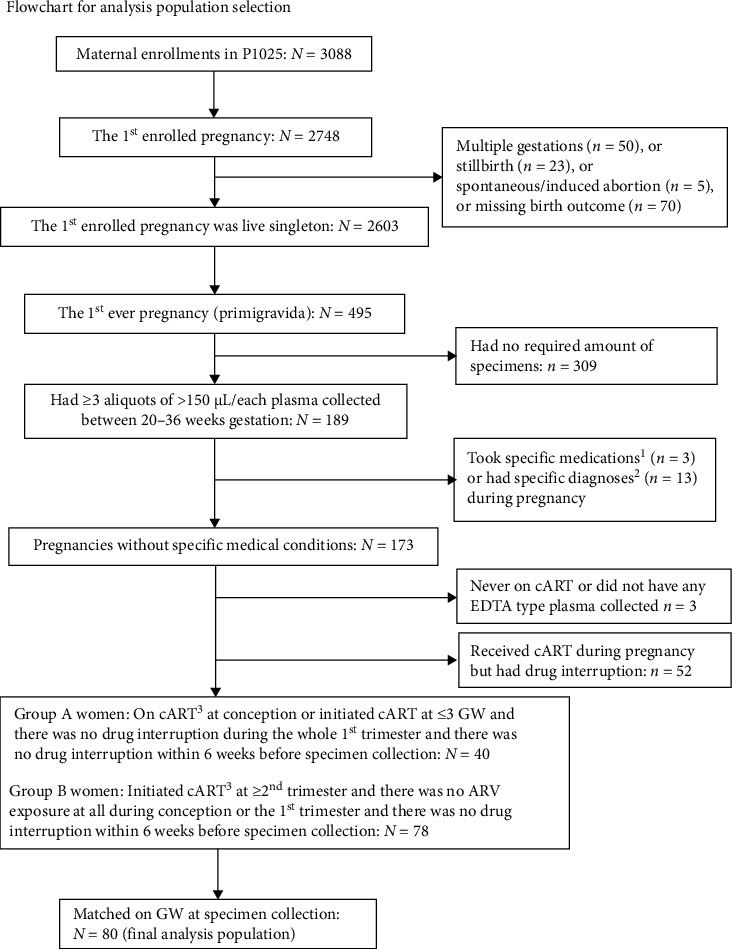
Selection of study samples. A total of 80 women were included in the analysis; 40 were on ART at conception and 40 initiated ART at ≥ the second trimester. The flowchart represents the selection process including exclusion inclusion criteria. ^1^Medications excluded: isoniazid, rifampicin, interferon alfa-2b, aspirin, methylprednisolone, solumedrom, mineralocorticoids, prednisolone, prednisone, hydrocortisone IV, and plaquenil sulfate. ^2^Diagnoses excluded: chronic, genetic, untreatable conditions with a systemic impact on the immune system during pregnancy; and concurrent conditions of transient, non-chronic nature that have a systemic impact on the immune system at the time of plasma sample collection. ^3^cART: combination ART including ≥3 drugs.

A total of 495 women enrolled with the first live birth singleton pregnancy, of whom 173 were eligible for selection into the two matched groups. Importantly, PPWH with specific preexisting conditions that would be expected to significantly alter the immune system or with diagnosis of inflammatory disorders at the time of sample collection were not included (Figure 1). This resulted in 80 women (40 were on ART at conception and 40 initiated ART at ≥ the second trimester) included in this analysis (Figure 1). All specimens were from GW 20–36 and were matched for GW by design. As shown in the Table 1, the study groups were also well balanced for age, race, height, and prepregnancy weight. The women on ART at conception were more likely to have CD4 count <350 cells/mm^3^ compared to women who initiated ART in the second trimester or later (34% versus 11%). Of note, there was no difference in perinatal outcomes between the groups, including the frequencies of preterm births, very preterm births, HDP, and PE (Table 1) and there were no intrauterine growth restriction (IUGR) diagnoses in either group. The vast majority of PPWH (85%) were on PI regimens (at least one PI among the ARV) at the time of conception. However, at the time of sample collection PI regimens were balanced between the groups (Supporting Information Table S1).

### 3.2. No Difference in Soluble Markers Profile in Plasma

**Figure 2:**
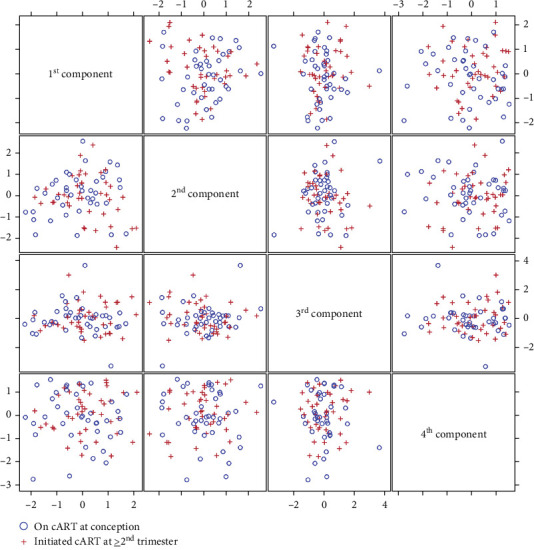
Principal component analysis (PCA) for soluble factors in plasma. PCA of the log-transformed plasma analytes was conducted to identify the underline construct of the analytes. Pairwise PC scores by group (blue = on ART at conception; red = ART started in the second trimester) are shown for the first four PCs, which explained 70% of the data variability.

Plasma was used to monitor soluble factors including chemokines, cytokines, angiogenic factors as well as factors with a known association with PE (i.e., PlGF and sFlt-1). For this analysis, we used both the Luminex and the Mesoscale platform (MSD) with custom multiplex panels that we had previously validated for sensitivity and accuracy on a different set of samples. No significant differences were noted when each analyte was compared directly between the two groups (Supporting Information Table S2). Moreover, PCA analysis of all the analytes did not lead to clear separation between the groups, suggesting that the overall plasma factors profile was similar between the groups (Figure 2 and Supporting Information Tables S3 and S4). Since adherence or resistance issues may have impacted the efficacy of the ARV regimen leading to detectable plasma viral loads and/or low CD4 counts, we performed a subset analysis excluding samples from PPWH that were viremic (>400 copies/mL) or had low CD4 counts (<350 cells/uL), because of their known heighted inflammatory profile. However, no significant differences were noted in this subset analysis (Table 2).

### 3.3. ART at Conception Leads to Lower Peripheral CD49a^+^ NK Cells

**Figure 3:**
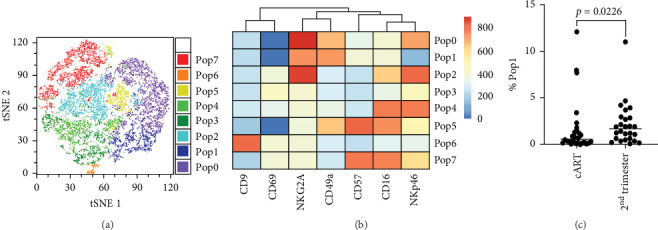
Higher frequency of NKG2A^+^ CD49a^+^ NK cells in PWLH on ART at conception. tSNE plot colored based on FlowSOM populations (A). Heat map of MFI for each marker in the NK panel in each FlowSOM population (B). Direct comparison of the frequency of Pop1 (NKG2A^+^ CD49a^+^ NK cells) between the two groups (C).

To better understand the immune environment associated with ART at conception compared to immune environment in PPWH who started ART in the second trimester, we performed an extensive phenotypic analysis by flow cytometry. We used four different panels encompassing markers of T cell subsets, myeloid cells, and NK cells (Supporting Information Table S5). Upon classical analysis including manual gating of specific subsets of interest, we found few differences. Memory CD4^+^ T cells (CD45RA^−^ within CD4^+^ T cells) and CD16^+^ NKG2A^−^ cytotoxic T cells were higher in the ART at conception group compared to the ART in second trimester group (*p*=0.06 in an analysis not adjusted for multiple comparisons; Supporting Information Table S6). Moreover, the ART at conception group had lower NKG2A^+^ NK cells, known to be tolerogenic [35] (unadjusted *p*=0.05) and CXCR3^+^ CCR6^+^ double positive cells within effector memory (CD45RA^−^ CD62L^−^) CD4^+^ T cells (unadjusted *p*=0.06; Supporting Information Table S6). Similar results were observed in the subset analysis excluding data from women with viremia and low CD4 counts Supporting Information Table S7). However, upon reanalysis of the data with an unsupervised clustering algorithm (FlowSOM), we identified eight distinct populations within the NK panel with differential expression of markers of NK activation, cytotoxicity, and tolerance (Figure 3A,B). Upon direct comparison of the frequencies of each population between the groups, we found that the ART at conception group had a significantly lower frequency of an NK subset expressing high levels of CD49a and medium level of NKG2A (Figure 3C). This confirms the lower level of NKG2A^+^ cells noticed with classical analysis, while adding the new insight of high CD49a expression.

### 3.4. Higher Transcriptional Levels of SERPINB2 in PBMC of PPWH on ART at Conception

**Figure 4:**
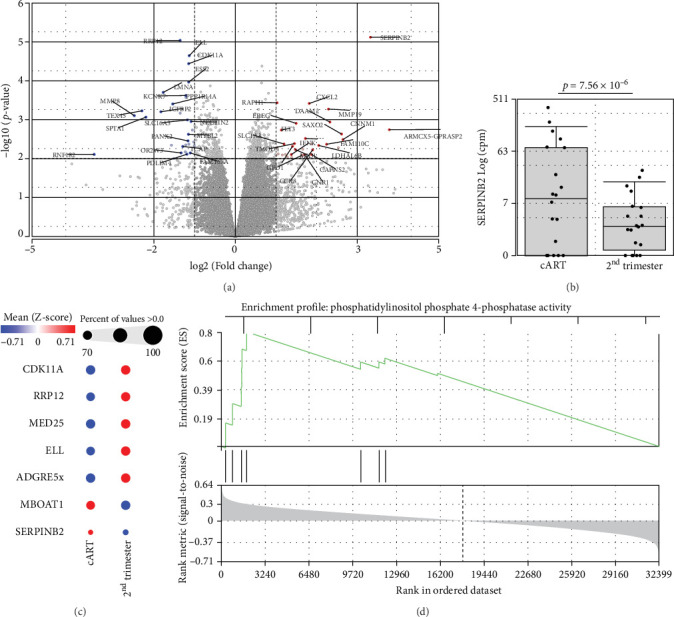
SERPINB2 expression is higher in the PBMC from the ART at conception group. **(**A) volcano plot of differentially expressed genes (DEGs) in bulk RNAseq analysis of PBMC from the ART at conception group compared to the second trimester group. Cutoffs at *p*  < 0.01 and log_2_ (FC) = 1 are shown. (B) direct comparisons of SERPINB2 counts in the two groups. (C) significantly different genes obtained by DESeq2 (FDR  < 0.1; log_2_FC = 1) in the T cell subset are shown with color proportional to *Z*-scores. (D) GSEA enrichment plot with FDR  < 0.1 (FDR = 0.02) is shown.

To get further insight into the immune environment of PPWH on ART at conception compared with PPWH who started ART in the second trimester, we performed bulk RNAseq of biobanked PBMC from a subset of 40 PPWH (20 samples per group matched for GW). Despite some heterogeneity in sample collection and storage, we identified 489 DEGs with a log_2_ (fold change [FC]) = 1 (Figure 4A) and 63 DEGs with log_2_ (FC) = 2. Interestingly, among the DEG, SERINB2 stands out as very highly upregulated in the ART at conception group compared to PBMC from PPWH that started ART in the second trimester (Figure 4A,B). SerpinB2 encodes for the Plasminogen activator inhibitor type-2 (PAI-2), a factor highly expressed during pregnancy [36], but also upregulated during inflammation [37] Upon filtering for genes with a highly significant FDR *q* <0.1 and log_2_ (FC) = 2, we obtained a list of seven DEGs (Figure 4C). Interestingly three of the five DEGs with lower expression in the samples from the ART at conception group, RRP12, ELL, and MED25 regulate cellular transcription (ribosomal function, elongation, and transcriptional regulation, respectively) [38–40]. Of the remaining downregulated genes, CDK11A is a critical cell cycle modulator and ADGRE5 is an important modulator of cell adhesion and migration [41, 42]. MBOAT1 is the only other gene enriched in the ART at conception group. It is involved in lipid metabolism and has a role in the biosynthesis of phosphatidylinositol [43]. Indeed, the most highly enriched gene ontology pathway and the only significantly enriched pathway with an FDR <0.1 was phosphatidylinositol phosphate 4-phosphatase (PI[4]P) activity (Figure 4D). A pathway involved in the activity of (PI[4]P) an enzyme that plays a crucial role in cellular signaling and lipid metabolism [44].

### 3.5. Discussion

PE is a leading cause of maternal and neonatal morbidity and mortality [45–47]. Although its etiology is likely heterogeneous, the disease begins with a dysregulation of trophoblast invasion and placental development, often driven by immune dysfunction and altered immune responses to trophoblast invasion [24]. The impact of ART at conception on placental development is still vastly understudied. Overwhelming evidence currently support the start of ART as soon as possible also during pregnancy. The use of ART at the time at conception has been shown to lead to positive maternal and fetal outcomes compared to no ART use [1].

Yet current treatment guidelines make comparisons of the impact of ART use at the time of conception with ART started later in pregnancy challenging. Hence, the biobanked specimens from the IMPAACT P1025 study were vital to making this comparison. The P1025 study included collection of samples from both ART-treated and ART-naïve PPWH. Because of the availability of several specimens, we could use relatively stringent inclusion and inclusion criteria to investigate the difference in immunological environment in the blood of women on ART at conception compared to women that started ART in the second trimester. Importantly, we could exclude inflammatory conditions that would have masked any difference.

Upon analysis of soluble factors, including cytokines and factors associated with PE development, we did not observe any difference between the groups. Although we only used ethylenediaminetetraacetic acid (EDTA)-derived plasma, we cannot exclude a high degree of variability in specimens, collection, and storage among the different sites. This variability may have been further increased by shipping to a central storage location and very long storage of over a decade from the time of collection. All these factors may have impaired our ability to detect differences in the soluble factors that we were interested in examining, including cytokines and growth factors, because of their known temperature/storage sensitivity [48, 49].

In contrast, PBMC sensitivity to collection and storage condition may explain the high variability in cell viability, but it likely did not substantially alter the cell phenotype. Hence, we were able to detect at least one important difference between the groups in the frequency of uterine CD49a^+^ NKG2A^+^ NK cells in the periphery. This very small cell subset may be precursor of tissue resident CD49^+^ NK cells [50], known to populate the uterine tissue in nonpregnant women and the decidua especially in early stages of pregnancy. These CD49^+^ NK cells have been shown to play a critical role during early placenta development in maintaining a tolerance at the fetal-maternal interface [51, 52], promote decidual vascularization, spiral artery formation and extravillous trophoblast invasion [53, 54]. The CD49^+^ NK cells are mainly present in tissues, but they can also be found in small percentage in blood, likely enroute to the tissues [50]. This finding, together with a tendency toward higher memory T cells (CD45RA^−^) and higher CD16^+^ NKG2A^−^ cytotoxic T cells in the ART at conception group compared to the ART in second trimester group suggests an imbalance in NK subsets in the ART at conception group that may reflect similar dysregulation at the level of the placental tissue.

Moreover, intriguingly, we found an upregulation of SERINB2 transcripts in the ART at conception group. SERPINE2 also known as plasminogen activator inhibitor type 2 (PAI-2), is a protein that plays a role in the regulation of fibrinolysis [55]. Although it is elevated systemically during inflammation in nonpregnant individuals [36], SERPINB2 is highly expressed in the human placenta throughout pregnancy with some evidence demonstrating it has a key role in the implantation process [56] Elevated levels of PAI-2 in pregnancy are associated with the suppression of fibrinolysis, helping to maintain a balance in the coagulation system and preventing excessive bleeding both during pregnancy and childbirth [57, 58]. However, since we detected higher levels of SERPINB2 in PBMC and not in the placental tissue, it is more likely an indicator of residual inflammation than produced as consequence of the pregnancy status. Yet transcriptional levels of this factor should be evaluated in non-pregnant individuals with similar ART timing to be certain that it is not the result also of the pregnancy.

This study has several important limitations. Its retrospective nature with the analysis of old specimens introduces a substantial degree of technical variability that cannot be estimated nor eliminated. Moreover, PPWH were treated with relatively old ARV regimens mostly including PIs. PI-based ART is considered as an alternative or second line therapy for most pregnant persons [59]. PI-use has been linked to higher rates of adverse birth outcomes and lower levels of progesterone in pregnant mice and women [60, 61]. Unfortunately, we did not have information regarding hormonal levels in the PPWH at the time of the specimen collection. Hence, we cannot determine whether we see an association between PI use at conception and frequency of CD49a^+^ NK or levels of SERPINB2 transcripts. Moreover, SERPINB2 enrichment in the ART at conception group could not be further validated by quantitative reverse transcription PCR (RT-qPCR) because of the lack of remaining PBMC or enough RNA left over. Finally, with only 40 samples/group and 20 samples/group in the RNAseq analysis, we were likely underpowered to see differences in this heterogeneous population.

Yet the observation of lower tolerogenic uterine CD49^+^ NK and higher SERPINB2 with their respective association with placental development and impaired pregnancy outcomes is intriguing considering no other difference was noted between the groups. Yet, larger studies powered to detect differences in perinatal outcomes are needed to confirm an association between these variables and placental insufficiencies relative to the timing of ART initiation.

The impact of novel ARV regiments that may further decrease inflammation [62] and of ARVs in absence of HIV infection (as during pre-exposure prophylaxis[PrEP]) at the time of conception will need to be investigated in larger, newer cohorts to determine the relevance of our findings to current therapeutic strategies.

## 4. Conclusions

Our work starts to uncover systemic mechanisms at play in PPWH associated with their use of ARVs that may have an impact on placental development and the course of the pregnancy. These mechanisms demand further investigation, and our observations require corroborations in other specimen cohorts and further validations.

## Figures and Tables

**Table 1:** Background characteristics by group.

Group of Women			
Characteristics	On cART at conception^a^ (*N* = 40)	Initiated cART at ≥ second trimester^b^ (*N* = 40)	*p*-Value
Gestational week at specimen collection			
20–23	4 (10%)	4 (10%)	1.00*⁣*^*∗*^
24–27	7 (18%)	7 (18%)	
28–31	9 (23%)	9 (23%)	
32	5 (13%)	5 (13%)	
33	2 (5%)	2 (5%)	
34	3 (8%)	3 (8%)	
35	4 (10%)	4 (10%)	
36	6 (15%)	6 (15%)	
Gestational week at specimen collection			
*N*	40	40	0.84*⁣*^*∗*^*⁣*^*∗*^
Mean (S.D.)	30 (5)	31 (4)	
Median (Q1, Q3)	32 (27, 35)	32 (27, 35)	
Min, max	20, 36	21, 36	
Age (years) at conception			
*N*	40	40	0.60*⁣*^*∗*^*⁣*^*∗*^
Mean (S.D.)	23.4 (6.0)	22.4 (4.9)	
Median (Q1, Q3)	21.4 (19.2, 25.9)	21.57 (18.9, 24.9)	
Min, max	15.6, 39.7	14.5, 36.6	
Race			
White/Other	18 (46%)	15 (38%)	0.65*⁣*^*∗*^
Black	21 (54%)	24 (62%)	
Unknown	1	1	
Hispanic or Latino			
No	29 (73%)	30 (75%)	1.00*⁣*^*∗*^
Yes	11 (28%)	10 (25%)	
Height (m)			
*N*	39	38	0.34*⁣*^*∗*^*⁣*^*∗*^
Mean (S.D.)	1.61 (0.07)	1.60 (0.07)	
Median (Q1, Q3)	1.63 (1.55, 1.68)	1.60 (1.57, 1.65)	
Min, max	1.36, 1.73	1.46, 1.75	
Prepregnancy weight (kg)			
*N*	32	31	0.14*⁣*^*∗*^*⁣*^*∗*^
Mean (S.D.)	72.6 (21.9)	84.3 (27.3)	
Median (Q1, Q3)	72.2 (55.6, 86.1)	74.3 (61.2, 105.8)	
Min, max	43.1, 129.0	50.9, 150.0	
Gestational week at initiating the first ARV during pregnancy, before specimen collection			
*N*	40	40	<0.001*⁣*^*∗*^*⁣*^*∗*^
Mean (S.D.)	0 (0)	19 (4)	
Median (Q1, Q3)		18 (16, 22)	
Min, max	0, 1	14, 26	
CD4 (cells/mm^3^) at the time of plasma specimen collection			
*N*	32	36	0.37*⁣*^*∗*^*⁣*^*∗*^
Mean (S.D.)	517 (285)	567 (239)	
Median (Q1, Q3)	505 (328, 680)	512 (399, 752)	
Min, max	25, 1,104	178, 1204	
CD4 at the time of plasma specimen collection: <350 cells/mm^3^			
Yes	11 (34%)	4 (11%)	0.04*⁣*^*∗*^
No	21 (66%)	32 (89%)	
Unknown	8	4	
RNA at the time of plasma specimen collection: ≥400 copies/mL			
Yes	6 (17%)	4 (11%)	0.51*⁣*^*∗*^
No	29 (83%)	32 (89%)	
Unknown	5	4	

*Note:* CD4 and viral load obtained within ±4 weeks and closest to the specimen collection.

^a^Women who were on cART (3+ drugs) at conception or initiated cART at ≤3 weeks gestation and there was no drug interruption during the whole first trimester and there was no drug interruption within 6 weeks before specimen collection.

^b^Women who initiated cART at ≥ the second trimester and there was no ARV exposure at all at conception or the first trimester and there was no drug interruption within 6 weeks before specimen collection.

*⁣* ^*∗*^Fisher's Exact Test.

*⁣* ^*∗*^ *⁣* ^*∗*^Wilcoxon Test.

**Table 2:** Concentrations of angiogenic and inflammatory biomarkers by ARV group, among women with VL <400 copies/mL and CD4 count ≥350 cells/mm.

Biomarker^a^ (pg/mL)	Women on cART at conception^b^	Women initiated cART at ≥ the second trimester^c^
TNF-α		
Mean (S.D.)	26.84 (30.08)	21.36 (9.62)
Median (Q1, Q3)	16.53 (10.47, 29.28)	19.42 (14.20, 25.68)
Min, max	6.47, 135.38	7.48, 47.04
IFN-γ		
Mean (S.D.)	56.91 (108.45)	42.11 (46.74)
Median (Q1, Q3)	22.95 (15.18, 55.16)	25.97 (16.53, 46.30)
Min, max	4.08, 465.63	5.11, 201.38
IL-4		
Mean (S.D.)	0.80 (0.99)	0.94 (1.31)
Median (Q1, Q3)	0.47 (0.16, 0.56)	0.40 (0.17, 1.02)
Min, max	0.05, 3.53	0.03, 5.74
IL-6		
Mean (S.D.)	45.90 (61.15)	33.82 (25.41)
Median (Q1, Q3)	28.70 (18.46, 52.79)	29.44 (12.37, 47.37)
Min, max	8.84, 272.26	1.44, 90.55
IL-10		
Mean (S.D.)	3.33 (3.87)	4.27 (5.29)
Median (Q1, Q3)	1.63 (0.86, 2.80)	2.01 (1.16, 6.15)
Min, max	0.49, 12.55	0.32, 23.82
IL-15		
Mean (S.D.)	37.05 (29.16)	30.46 (15.71)
Median (Q1, Q3)	32.18 (19.45, 44.02)	27.41 (18.65, 40.19)
Min, max	10.10, 140.27	8.18, 66.49
TGF-β		
Mean (S.D.)	1266.07 (852.28)	1281.50 (885.43)
Median (Q1, Q3)	953.28 (435.33, 1954.78)	1213.32 (453.72, 2200.95)
Min, max	214.10, 2559.36	71.97, 2630.08
IL-1β		
Mean (S.D.)	15.18 (11.48)	14.89 (12.37)
Median (Q1, Q3)	12.41 (5.67, 21.64)	9.45 (7.26, 24.30)
Min, max	3.74, 39.67	1.02, 56.08
IL-18		
Mean (S.D.)	130.65 (106.29)	148.99 (121.52)
Median (Q1, Q3)	102.69 (59.29, 134.88)	115.50 (76.59, 172.64)
Min, max	24.75, 372.90	28.38, 509.79
IL-5		
Mean (S.D.)	5.60 (6.50)	6.59 (8.73)
Median (Q1, Q3)	3.45 (1.31, 5.40)	3.33 (1.66, 7.66)
Min, max	0.69, 22.79	0.03, 35.82
IL-13		
Mean (S.D.)	15.31 (16.84)	11.80 (9.39)
Median (Q1, Q3)	11.16 (7.80, 16.30)	9.27 (4.05, 13.99)
Min, max	3.06, 76.12	2.38, 35.59
CCL-11		
Mean (S.D.)	7.67 (4.02)	7.16 (2.52)
Median (Q1, Q3)	6.83 (5.88, 8.19)	6.66 (5.50, 7.89)
Min, max	2.80, 20.06	3.68, 15.93
RANTES		
Mean (S.D.)	28.31 (11.90)	33.94 (23.87)
Median (Q1, Q3)	28.92 (18.43, 35.84)	30.58 (23.51, 36.89)
Min, max	12.25, 53.72	13.26, 148.05
MIP-1α		
Mean (S.D.)	8.88 (12.32)	8.19 (6.56)
Median (Q1, Q3)	4.44 (3.99, 8.59)	6.07 (4.13, 8.58)
Min, max	1.86, 54.53	1.56, 27.31
MCP-1		
Mean (S.D.)	16.05 (6.18)	16.63 (6.60)
Median (Q1, Q3)	14.89 (12.18, 22.74)	16.30 (12.06, 19.65)
Min, max	5.34, 24.99	8.42, 38.91
IP-10		
Mean (S.D.)	12.72 (7.74)	16.34 (11.65)
Median (Q1, Q3)	10.94 (7.35, 15.08)	11.98 (10.54, 15.85)
Min, max	2.83, 34.36	5.33, 59.50
P-Selectin		
Mean (S.D.)	71,484.52 (71,091.59)	59,797.54 (55,513.75)
Median (Q1, Q3)	52,107.29 (32,994.31, 77,041.90)	44,245.80 (28,841.35, 62,917.75)
Min, max	23,364.24, 322,494.66	18,873.73, 285,446.37
IL-12p70		
Mean (S.D.)	3.95 (4.75)	4.89 (6.84)
Median (Q1, Q3)	2.28 (0.82, 3.81)	2.53 (1.09, 6.37)
Min, max	0.22, 16.33	0.13, 30.25
VEGF-A		
Mean (S.D.)	39.70 (23.30)	38.58 (22.79)
Median (Q1, Q3)	32.57 (26.01, 47.92)	35.06 (23.97, 41.85)
Min, max	1.49, 95.80	5.86, 114.31
GM-CSF		
Mean (S.D.)	0.68 (0.71)	0.93 (1.20)
Median (Q1, Q3)	0.54 (0.19, 0.71)	0.48 (0.20, 1.61)
Min, max	0.03, 2.13	0.05, 5.86
IL-17α		
Mean (S.D.)	8.07 (9.54)	11.70 (17.34)
Median (Q1, Q3)	5.05 (2.12, 8.74)	5.11 (2.82, 9.23)
Min, max	0.41, 29.90	0.63, 83.57
E-selectin		
Mean (S.D.)	8592.58 (2327.85)	9530.82 (3853.67)
Median (Q1, Q3)	8598.66 (6994.65, 9758.39)	9,076.57 (7126.72, 10,374.48)
Min, max	4494.24, 13,428.94	4539.42, 24,582.15
SDF-1α		
Mean (S.D.)	477.50 (404.76)	467.25 (268.32)
Median (Q1, Q3)	402.84 (187.33, 603.10)	395.75 (290.87, 582.54)
Min, max	35.86, 1733.17	98.83, 1416.42
ICAM-1		
Mean (S.D.)	74,565.02 (89,425.18)	80,667.46 (116,802.18)
Median (Q1, Q3)	53,248.27 (34,250.09, 57,391.92)	47,457.71 (33,258.66, 74,228.98)
Min, max	22,706.77, 398,212.68	6024.70, 636,124.51
Ang-1		
Mean (S.D.)	3941.09 (3492.67)	3637.34 (3553.47)
Median (Q1, Q3)	3276.24 (1207.25, 5466.70)	1,909.04 (809.63, 5888.80)
Min, max	63.29, 12,230.76	108.25, 13,201.42
Ang-2		
Mean (S.D.)	8788.63 (5567.40)	9225.39 (5279.93)
Median (Q1, Q3)	8456.61 (4744.40, 10,082.14)	9536.82 (4713.66, 12,094.78)
Min, max	794.18, 20,047.23	1,345.07, 22,791.20
PlGF		
Mean (S.D.)	170.45 (192.23)	182.18 (135.94)
Median (Q1, Q3)	121.33 (33.36, 161.73)	135.59 (80.03, 263.26)
Min, max	6.31, 674.44	23.67, 538.81
sFlt-1		
Mean (S.D.)	1261.28 (995.73)	1084.81 (679.48)
Median (Q1, Q3)	891.45 (593.70, 1445.62)	977.54 (577.70, 1490.77)
Min, max	168.34, 3737.01	266.51, 2750.59
Log(VEGF-A)/Log(sFlt-1)		
Mean (S.D.)	0.51 (0.16)	0.52 (0.10)
Median (Q1, Q3)	0.50 (0.44, 0.59)	0.53 (0.45, 0.57)
Min, max	0.06, 0.75	0.26, 0.75
Log(sFlt-1) /Log(PlGF)		
Mean (S.D.)	1.71 (0.75)	1.44 (0.38)
Median (Q1, Q3)	1.47 (1.26, 2.11)	1.30 (1.20, 1.63)
Min, max	0.79, 3.84	1.00, 2.49
Log(Ang-1) /Log(Ang-2)		
Mean (S.D.)	0.87 (0.14)	0.85 (0.15)
Median (Q1, Q3)	0.89 (0.75, 0.98)	0.87 (0.78, 0.94)
Min, max	0.62, 1.09	0.51, 1.10
Log(IL-15)/Log(IL-10)		
Mean (S.D.)	−6.37 (26.63)	10.71 (42.75)
Median (Q1, Q3)	1.54 (-8.09, 5.00)	1.94 (0.92, 4.75)
Min, max	−104.13, 13.10	−10.50, 230.00

*Note:* For five participants whose IL-6 concentration was not obtained and 13 participants whose IL-1RA concentration was not obtained because of the levels were below the LOD, the values of LOD (IL-6: 1.44 pg/mL; IL-1RA: 28.91 pg/mL) were used in the analysis.

^a^Wilcoxon rank-sum test for nontransformed concentrations; two-sample equal variance *t*-test for log_10_ transformed concentrations (log_10_ data not shown). *p*-values were omitted since all values were nonsignificant.

^b^PLWH with plasma viral load <400 copies/mL and CD4 counts >350 cells/uL *n* = 17.

^c^PLWH with plasma viral load <400 copies/mL and CD4 counts >350 cells/uL *n* = 29.

## Data Availability

The sequencing data discussed in this publication have been deposited in NCBI GEO and are accessible through GSE275120 https://www.ncbi.nlm.nih.gov/geo/query/acc.cgi?acc=GSE275120. All other data are included in the manuscript.

## Supporting Information

Supporting Information 1 Table S1: ARV regimens at conception and at time of specimen collection by ARV group.

Supporting Information 2 Table S2: Concentrations of angiogenic and inflammatory biomarkers by ARV group, among all women.

Supporting Information 3 Table S3: Eigenvalues of the correlation matrix for principal components for inflammatory biomarkers.

Supporting Information 4 Table S4: Rotated factor loading pattern of the first four principal components for inflammatory biomarkers.

Supporting Information 5 Table S5: List of antibodies for flow cytometry.

Supporting Information 6 Table S6: Immune phenotyping biomarker percentages by ARV group, within different parent markers, among all women.

Supporting Information 7 Table S7: Immune phenotyping biomarker percentages by ARV group, within different parent markers, among women with VL < 400 copies/mL and CD4 count ≥350 cells/mm^3^.
