# Supplementary material for: Antiretroviral Therapy at Conception Leads to Lower Peripheral CD49a+ NK Cells and Higher SERPINB2
Source: J Immunol Res. 2025 May 21;2025:4771787. doi: 10.1155/jimr/4771787 (PMC12119168; doi:10.1155/jimr/4771787)
Supplement: Supporting Information 1 — Table S1: ARV regimens at conception and at time of specimen collection by ARV group. [file 4771787.f1.docx]

**Table S1: ARV regimens at conception and at time of specimen collection by ARV group**

|  | **ARV Regimen at Conception** |  | **ARV Regimen at Time of Specimen Collections** | |
| --- | --- | --- | --- | --- |
| **ARV Regimen** | **Women on cART at Conception¹ (N=40)** |  | **Women on cART at Conception (N=40)** | **Women Initiated cART at ≥ 2nd Trimester (N=40)** |
| 3tc,tdf,lpv | 2 (5%) |  | 1 (3%) | 0 (0%) |
| 3tc,tdf,nfv | 1 (3%) |  | 1 (3%) | 0 (0%) |
| 3tc,zdv,efv | 1 (3%) |  | 0 (0%) | 0 (0%) |
| 3tc,zdv,ftc,tdf,rtv,atv | 1 (3%) |  | 0 (0%) | 0 (0%) |
| 3tc,zdv,lpv | 4 (10%) |  | 8 (20%) | 12 (30%) |
| 3tc,zdv,nfv | 3 (8%) |  | 2 (5%) | 12 (30%) |
| 3tc,zdv,nvp | 2 (5%) |  | 2 (5%) | 4 (10%) |
| 3tc,zdv,rtv,atv | 1 (3%) |  | 1 (3%) | 0 (0%) |
| 3tc,zdv,rtv,fpv | 0 (0%) |  | 0 (0%) | 1 (3%) |
| 3tc,zdv,tdf,lpv | 1 (3%) |  | 1 (3%) | 0 (0%) |
| 3tc,zdv,tdf,rtv,fpv | 0 (0%) |  | 1 (3%) | 0 (0%) |
| abc,3tc,atv | 2 (5%) |  | 0 (0%) | 0 (0%) |
| abc,3tc,efv,atv | 1 (3%) |  | 0 (0%) | 0 (0%) |
| abc,3tc,fpv | 1 (3%) |  | 1 (3%) | 0 (0%) |
| abc,3tc,lpv | 1 (3%) |  | 2 (5%) | 0 (0%) |
| abc,3tc,rtv,atv | 0 (0%) |  | 1 (3%) | 0 (0%) |
| abc,3tc,zdv | 1 (3%) |  | 2 (5%) | 7 (18%) |
| abc,3tc,zdv,lpv | 1 (3%) |  | 1 (3%) | 1 (3%) |
| abc,3tc,zdv,rtv,atv | 0 (0%) |  | 2 (5%) | 0 (0%) |
| abc,3tc,zdv,tdf | 1 (3%) |  | 1 (3%) | 0 (0%) |
| d4t,3tc,lpv | 2 (5%) |  | 2 (5%) | 0 (0%) |
| d4t,3tc,nvp | 1 (3%) |  | 0 (0%) | 0 (0%) |
| ddi,3tc,zdv,lpv | 0 (0%) |  | 1 (3%) | 0 (0%) |
| ddi,3tc,zdv,tdf,sqv,lpv | 1 (3%) |  | 0 (0%) | 0 (0%) |
| ddi,zdv,rtv,atv | 0 (0%) |  | 0 (0%) | 1 (3%) |
| ftc,tdf,atv | 1 (3%) |  | 1 (3%) | 0 (0%) |
| ftc,tdf,lpv | 2 (5%) |  | 1 (3%) | 0 (0%) |
| ftc,tdf,rtv,atv | 7 (18%) |  | 4 (10%) | 1 (3%) |
| ftc,tdf,rtv,drv,ral | 1 (3%) |  | 1 (3%) | 0 (0%) |
| ftc,tdf,rtv,fpv | 0 (0%) |  | 1 (3%) | 0 (0%) |
| ftc,tdf,rtv,sqv | 0 (0%) |  | 0 (0%) | 1 (3%) |
| tdf,efv,rtv,atv | 1 (3%) |  | 0 (0%) | 0 (0%) |
| zdv,ftc,tdf,rtv,atv | 0 (0%) |  | 1 (3%) | 0 (0%) |
| zdv,tdf,lpv | 0 (0%) |  | 1 (3%) | 0 (0%) |

*¹ 1 woman initiated the cART at 1 week gestation.*
