# Supplementary material for: Antiretroviral Therapy at Conception Leads to Lower Peripheral CD49a+ NK Cells and Higher SERPINB2
Source: J Immunol Res. 2025 May 21;2025:4771787. doi: 10.1155/jimr/4771787 (PMC12119168; doi:10.1155/jimr/4771787)
Supplement: Supporting Information 2 — Table S2: Concentrations of angiogenic and inflammatory biomarkers by ARV group, among all women. [file 4771787.f2.docx]

**Table S2: Concentrations of angiogenic and inflammatory biomarkers by ARV group, among all women**

| **Biomarker (pg/mL)** |  | **Women on ART at Conception¹** | **Women initiated ART at ≥ the 2nd trimester²** | **P-Value ³** |
| --- | --- | --- | --- | --- |
| Log10 TNF alpha | Mean (s.d.) | 1.30 (0.29) | 1.32 (0.22) | 0.72 |
|  | Median (Q1, Q3) | 1.29 (1.14, 1.47) | 1.33 (1.18, 1.48) |  |
|  | Min, Max | 0.53, 2.13 | 0.87, 1.90 |  |
| Log10 IFN gamma | Mean (s.d.) | 1.42 (0.42) | 1.54 (0.44) | 0.22 |
|  | Median (Q1, Q3) | 1.35 (1.15, 1.75) | 1.50 (1.23, 1.86) |  |
|  | Min, Max | 0.61, 2.67 | 0.71, 2.63 |  |
| Log10 IL-4 | Mean (s.d.) | -0.48 (0.55) | -0.36 (0.56) | 0.33 |
|  | Median (Q1, Q3) | -0.47 (-0.90, -0.07) | -0.39 (-0.79, 0.12) |  |
|  | Min, Max | -1.48, 0.55 | -1.50, 0.76 |  |
| Log10 IL-6 | Mean (s.d.) | 1.44 (0.44) | 1.38 (0.50) | 0.57 |
|  | Median (Q1, Q3) | 1.52 (1.26, 1.73) | 1.52 (1.15, 1.68) |  |
|  | Min, Max | 0.16, 2.44 | 0.16, 2.18 |  |
| Log10 IL-10 | Mean (s.d.) | 0.25 (0.45) | 0.41 (0.45) | 0.12 |
|  | Median (Q1, Q3) | 0.20 (-0.12, 0.53) | 0.37 (0.10, 0.75) |  |
|  | Min, Max | -0.45, 1.10 | -0.50, 1.38 |  |
| Log10 IL-15 | Mean (s.d.) | 1.47 (0.24) | 1.44 (0.26) | 0.52 |
|  | Median (Q1, Q3) | 1.51 (1.35, 1.62) | 1.47 (1.30, 1.62) |  |
|  | Min, Max | 0.90, 2.15 | 0.79, 1.90 |  |
| Log10 TGF beta | Mean (s.d.) | 2.89 (0.44) | 2.98 (0.38) | 0.32 |
|  | Median (Q1, Q3) | 2.95 (2.53, 3.29) | 3.09 (2.67, 3.33) |  |
|  | Min, Max | 1.70, 3.42 | 1.86, 3.42 |  |
| Log10 IL-1 beta | Mean (s.d.) | 1.05 (0.37) | 1.04 (0.43) | 0.90 |
|  | Median (Q1, Q3) | 1.14 (0.79, 1.30) | 1.07 (0.87, 1.39) |  |
|  | Min, Max | -0.05, 1.64 | 0.01, 1.87 |  |
| Log10 IL-18 | Mean (s.d.) | 2.03 (0.31) | 2.08 (0.32) | 0.52 |
|  | Median (Q1, Q3) | 1.99 (1.84, 2.16) | 2.07 (1.88, 2.34) |  |
|  | Min, Max | 1.39, 2.74 | 1.45, 2.71 |  |
| Log10 IL-5 | Mean (s.d.) | 0.34 (0.64) | 0.50 (0.62) | 0.28 |
|  | Median (Q1, Q3) | 0.43 (0.04, 0.80) | 0.54 (0.20, 0.98) |  |
|  | Min, Max | -1.59, 1.36 | -1.60, 1.55 |  |
| Log10 IL-13 | Mean (s.d.) | 1.05 (0.32) | 0.97 (0.38) | 0.33 |
|  | Median (Q1, Q3) | 1.05 (0.88, 1.23) | 1.00 (0.68, 1.21) |  |
|  | Min, Max | 0.23, 1.88 | -0.03, 1.81 |  |
| Log10 CCL-11 | Mean (s.d.) | 0.86 (0.17) | 0.84 (0.14) | 0.55 |
|  | Median (Q1, Q3) | 0.88 (0.77, 0.99) | 0.82 (0.75, 0.92) |  |
|  | Min, Max | 0.45, 1.30 | 0.57, 1.20 |  |
| Log10 RANTES | Mean (s.d.) | 1.44 (0.20) | 1.48 (0.19) | 0.36 |
|  | Median (Q1, Q3) | 1.47 (1.27, 1.57) | 1.48 (1.38, 1.55) |  |
|  | Min, Max | 1.04, 1.83 | 1.12, 2.17 |  |
| Log10 MIP-1 alpha | Mean (s.d.) | 0.80 (0.30) | 0.86 (0.30) | 0.44 |
|  | Median (Q1, Q3) | 0.75 (0.62, 0.94) | 0.79 (0.63, 1.05) |  |
|  | Min, Max | 0.27, 1.74 | 0.19, 1.45 |  |
|  | Min, Max | 5.34, 40.90 | 8.42, 40.11 |  |
| Log10 MCP-1 | Mean (s.d.) | 1.20 (0.20) | 1.20 (0.17) | 0.98 |
|  | Median (Q1, Q3) | 1.22 (1.05, 1.36) | 1.20 (1.09, 1.32) |  |
|  | Min, Max | 0.73, 1.61 | 0.93, 1.60 |  |
| Log10 IP-10 | Mean (s.d.) | 1.09 (0.30) | 1.16 (0.24) | 0.21 |
|  | Median (Q1, Q3) | 1.08 (0.89, 1.27) | 1.10 (1.02, 1.35) |  |
|  | Min, Max | 0.45, 2.16 | 0.73, 1.77 |  |
| Log10 P-Selectin | Mean (s.d.) | 4.69 (0.29) | 4.68 (0.26) | 0.77 |
|  | Median (Q1, Q3) | 4.67 (4.44, 4.90) | 4.66 (4.47, 4.81) |  |
|  | Min, Max | 4.19, 5.51 | 4.28, 5.46 |  |
| Log10 IL-12p70 | Mean (s.d.) | 0.20 (0.58) | 0.42 (0.59) | 0.10 |
|  | Median (Q1, Q3) | 0.23 (-0.12, 0.60) | 0.41 (0.04, 0.87) |  |
|  | Min, Max | -0.98, 1.21 | -0.88, 1.55 |  |
| Log10 VEGF-A | Mean (s.d.) | 1.63 (0.38) | 1.51 (0.26) | 0.12 |
|  | Median (Q1, Q3) | 1.62 (1.47, 1.76) | 1.54 (1.38, 1.63) |  |
|  | Min, Max | 0.17, 2.64 | 0.77, 2.06 |  |
| Log10 GM-CSF | Mean (s.d.) | -0.52 (0.55) | -0.32 (0.50) | 0.08 |
|  | Median (Q1, Q3) | -0.46 (-0.87, -0.15) | -0.32 (-0.73, 0.08) |  |
|  | Min, Max | -1.70, 0.33 | -1.28, 0.77 |  |
| Log10 IL-17a | Mean (s.d.) | 0.58 (0.51) | 0.72 (0.53) | 0.24 |
|  | Median (Q1, Q3) | 0.54 (0.11, 0.93) | 0.73 (0.31, 1.08) |  |
|  | Min, Max | -0.39, 1.50 | -0.34, 1.92 |  |
| Log10 E-selectin | Mean (s.d.) | 3.95 (0.14) | 3.93 (0.16) | 0.62 |
|  | Median (Q1, Q3) | 3.96 (3.84, 4.05) | 3.95 (3.85, 4.00) |  |
|  | Min, Max | 3.65, 4.18 | 3.55, 4.39 |  |
| Log10 SDF-1a | Mean (s.d.) | 2.65 (0.44) | 2.60 (0.23) | 0.56 |
|  | Median (Q1, Q3) | 2.65 (2.40, 2.84) | 2.60 (2.47, 2.77) |  |
|  | Min, Max | 1.56, 4.55 | 2.00, 3.15 |  |
| Log10 ICAM-1 | Mean (s.d.) | 4.76 (0.30) | 4.76 (0.34) | 0.98 |
|  | Median (Q1, Q3) | 4.73 (4.58, 4.91) | 4.74 (4.56, 4.96) |  |
|  | Min, Max | 4.16, 5.60 | 3.78, 5.80 |  |
| Log10 Ang-1 | Mean (s.d.) | 3.16 (0.67) | 3.29 (0.55) | 0.36 |
|  | Median (Q1, Q3) | 3.22 (2.74, 3.72) | 3.29 (2.98, 3.74) |  |
|  | Min, Max | 1.63, 4.10 | 1.99, 4.12 |  |
| Log10 Ang-2 | Mean (s.d.) | 3.88 (0.31) | 3.93 (0.29) | 0.46 |
|  | Median (Q1, Q3) | 3.93 (3.68, 4.06) | 4.04 (3.72, 4.13) |  |
|  | Min, Max | 2.90, 4.36 | 3.13, 4.36 |  |
| Log10 PlGF | Mean (s.d.) | 2.11 (0.46) | 2.13 (0.39) | 0.88 |
|  | Median (Q1, Q3) | 2.17 (1.93, 2.39) | 2.12 (1.89, 2.41) |  |
|  | Min, Max | 0.80, 2.83 | 1.37, 2.91 |  |
| Log10 sFlt-1 | Mean (s.d.) | 2.93 (0.32) | 2.91 (0.29) | 0.70 |
|  | Median (Q1, Q3) | 2.96 (2.72, 3.16) | 2.96 (2.71, 3.14) |  |
|  | Min, Max | 2.19, 3.57 | 2.35, 3.44 |  |
| Ratio of VEGF-A/sFlt-1 | N | 40 | 40 | 0.27 |
|  | Mean (s.d.) | 0.08 (0.10) | 0.05 (0.04) |  |
|  | Median (Q1, Q3) | 0.05 (0.03, 0.10) | 0.05 (0.03, 0.07) |  |
|  | Min, Max | 0.00, 0.56 | 0.01, 0.21 |  |
| Log10 ratio of VEGF-A/sFlt-1 | Mean (s.d.) | -1.30 (0.45) | -1.39 (0.35) | 0.33 |
|  | Median (Q1, Q3) | -1.27 (-1.56, -0.99) | -1.34 (-1.61, -1.14) |  |
|  | Min, Max | -2.52, -0.25 | -2.23, -0.67 |  |
| Ratio of sFlt-1/PlGF | N | 40 | 40 | 0.80 |
|  | Mean (s.d.) | 21.67 (41.29) | 13.68 (23.51) |  |
|  | Median (Q1, Q3) | 5.44 (2.32, 12.38) | 4.49 (2.87, 11.92) |  |
|  | Min, Max | 0.25, 187.10 | 1.01, 113.53 |  |
| Log10 ratio of sFlt-1/PlGF | Mean (s.d.) | 0.82 (0.66) | 0.78 (0.51) | 0.76 |
|  | Median (Q1, Q3) | 0.73 (0.36, 1.09) | 0.65 (0.46, 1.07) |  |
|  | Min, Max | -0.60, 2.27 | 0.01, 2.06 |  |
| Ratio of Ang-1/Ang-2 | N | 40 | 40 | 0.47 |
|  | Mean (s.d.) | 0.59 (0.98) | 0.48 (0.57) |  |
|  | Median (Q1, Q3) | 0.19 (0.07, 0.85) | 0.31 (0.09, 0.58) |  |
|  | Min, Max | 0.01, 4.30 | 0.00, 2.44 |  |
| Log10 ratio of Ang-1/Ang-2 | Mean (s.d.) | -0.72 (0.70) | -0.64 (0.62) | 0.60 |
|  | Median (Q1, Q3) | -0.72 (-1.19, -0.07) | -0.50 (-1.03, -0.24) |  |
|  | Min, Max | -2.13, 0.63 | -2.37, 0.39 |  |
| Ratio of IL-15/IL-10 | N | 40 | 40 | 0.16 |
|  | Mean (s.d.) | 24.59 (20.76) | 19.86 (21.69) |  |
|  | Median (Q1, Q3) | 18.31 (9.44, 34.13) | 15.61 (4.56, 25.53) |  |
|  | Min, Max | 1.63, 82.71 | 0.46, 105.46 |  |
| Log10 ratio of IL-15/IL-10 | Mean (s.d.) | 1.23 (0.41) | 1.03 (0.55) | 0.08 |
|  | Median (Q1, Q3) | 1.26 (0.98, 1.53) | 1.19 (0.65, 1.41) |  |
|  | Min, Max | 0.21, 1.92 | -0.34, 2.02 |  |
|  |  |  |  |  |

*¹ Women who were on cART (3+ drugs) at conception or initiated cART at ≤ 3 weeks gestation AND there was no drug interruption during the whole 1st trimester AND there was no drug interruption within 6 weeks before specimen collection.*

*² Women who initiated cART at ≥ the 2nd trimester AND there was no ARV exposure at all at conception or the 1st trimester AND there was no drug interruption within 6 weeks before specimen collection.*

*³ Wilcoxon rank-sum test for non-transformed concentrations; two-sample equal-variance T test for log10 transformed concentrations.*

*For 5 participants whose IL-6 concentration was not obtained because of the levels were below the LOD, the values of LOD (1.44pg/mL) was used in the analysis.*
