# Supplementary material for: Antiretroviral Therapy at Conception Leads to Lower Peripheral CD49a+ NK Cells and Higher SERPINB2
Source: J Immunol Res. 2025 May 21;2025:4771787. doi: 10.1155/jimr/4771787 (PMC12119168; doi:10.1155/jimr/4771787)
Supplement: Supporting Information 3 — Table S3: Eigenvalues of the correlation matrix for principal components for inflammatory biomarkers. [file 4771787.f3.docx]

**Table S3: Eigenvalues of the correlation matrix for principal components for inflammatory biomarkers**

| **Order** | **Eigenvalue** | **% Variance Explained** | **Cumulative % Variance Explained** |
| --- | --- | --- | --- |
| 1 | 9.319 | 0.333 | 0.333 |
| 2 | 5.352 | 0.191 | 0.524 |
| 3 | 3.327 | 0.119 | 0.643 |
| 4 | 1.586 | 0.057 | 0.699 |
| 5 | 1.244 | 0.044 | 0.744 |
| 6 | 1.036 | 0.037 | 0.781 |
| 7 | 0.988 | 0.035 | 0.816 |
| 8 | 0.759 | 0.027 | 0.843 |
| 9 | 0.644 | 0.023 | 0.866 |
| 10 | 0.589 | 0.021 | 0.887 |
| 11 | 0.506 | 0.018 | 0.905 |
| 12 | 0.422 | 0.015 | 0.92 |
| 13 | 0.359 | 0.013 | 0.933 |
| 14 | 0.316 | 0.011 | 0.945 |
| 15 | 0.278 | 0.01 | 0.954 |
| 16 | 0.239 | 0.009 | 0.963 |
| 17 | 0.202 | 0.007 | 0.97 |
| 18 | 0.189 | 0.007 | 0.977 |
| 19 | 0.154 | 0.006 | 0.983 |
| 20 | 0.126 | 0.004 | 0.987 |
| 21 | 0.084 | 0.003 | 0.99 |
| 22 | 0.075 | 0.003 | 0.993 |
| 23 | 0.064 | 0.002 | 0.995 |
| 24 | 0.041 | 0.001 | 0.996 |
| 25 | 0.036 | 0.001 | 0.998 |
| 26 | 0.027 | 0.001 | 0.999 |
| 27 | 0.024 | 0.001 | 1 |
| 28 | 0.013 | 0 | 1 |
