# Supplementary material for: Antiretroviral Therapy at Conception Leads to Lower Peripheral CD49a+ NK Cells and Higher SERPINB2
Source: J Immunol Res. 2025 May 21;2025:4771787. doi: 10.1155/jimr/4771787 (PMC12119168; doi:10.1155/jimr/4771787)
Supplement: Supporting Information 4 — Table S4: Rotated factor loading pattern of the first four principal components for inflammatory biomarkers. [file 4771787.f4.docx]

**Table S4:** **Rotated factor loading pattern of the first 4 principal components for inflammatory biomarkers**

| **Biomarker** | **1st Component** | **2nd Component** | **3rd Component** | **4th Component** |
| --- | --- | --- | --- | --- |
| Log10 IL-4 | 0.957 | 0.043 | -0.098 | 0.015 |
| Log10 IL-5 | 0.949 | 0.064 | -0.067 | 0.022 |
| Log10 GM-CSF | 0.934 | 0.103 | -0.116 | 0.027 |
| Log10 IL-17a | 0.932 | 0.085 | -0.163 | 0.080 |
| Log10 IL-12p70 | 0.932 | 0.075 | -0.072 | 0.043 |
| Log10 IL-10 | 0.929 | 0.017 | -0.120 | 0.039 |
| Log10 Ang-1 | 0.836 | 0.081 | -0.059 | 0.005 |
| Log10 IFN gamma | 0.818 | -0.014 | -0.017 | 0.150 |
| Log10 TGF beta | 0.715 | 0.107 | 0.081 | -0.200 |
| Log10 IL-13 | -0.072 | 0.924 | -0.161 | 0.020 |
| Log10 IL-1 beta | 0.140 | 0.908 | -0.225 | 0.092 |
| Log10 IL-15 | 0.115 | 0.905 | -0.087 | 0.117 |
| Log10 IL-6 | 0.034 | 0.887 | -0.334 | 0.000 |
| Log10 TNF alpha | 0.309 | 0.838 | 0.059 | 0.107 |
| Log10 MCP-1 | 0.050 | 0.783 | 0.066 | -0.048 |
| Log10 CCL-11 | -0.061 | 0.629 | 0.392 | -0.228 |
| Log10 MIP-1 alpha | -0.105 | 0.602 | 0.297 | -0.079 |
| Log10 P-Selectin | 0.272 | 0.462 | 0.194 | 0.038 |
| Log10 VEGF-A | -0.238 | 0.189 | 0.748 | -0.201 |
| Log10 SDF-1a | -0.403 | 0.049 | 0.721 | -0.154 |
| Log10 IP-10 | 0.163 | -0.001 | 0.716 | -0.080 |
| Log10 IL-18 | 0.237 | 0.066 | 0.703 | 0.039 |
| Log10 E-selectin | -0.402 | -0.237 | 0.684 | 0.046 |
| Log10 RANTES | -0.530 | -0.153 | 0.613 | 0.020 |
| Log10 ICAM-1 | -0.409 | -0.185 | 0.478 | 0.162 |
| Log10 PlGF | 0.013 | 0.002 | -0.001 | 0.757 |
| Log10 Ang-2 | 0.177 | 0.140 | 0.058 | 0.727 |
| Log10 sFlt-1 | 0.108 | 0.050 | 0.210 | -0.545 |

*Varimax rotation method was used to obtain orthogonal components.*

*Loading coefficients ≥ 0.50 on a specific component were shown in red color.*
