# Supplementary material for: Antiretroviral Therapy at Conception Leads to Lower Peripheral CD49a+ NK Cells and Higher SERPINB2
Source: J Immunol Res. 2025 May 21;2025:4771787. doi: 10.1155/jimr/4771787 (PMC12119168; doi:10.1155/jimr/4771787)
Supplement: Supporting Information 5 — Table S5: List of antibodies for flow cytometry. [file 4771787.f5.docx]

**Table S5 List of antibodies for flow cytometry**

| **T PANEL** |  |  |  |
| --- | --- | --- | --- |
| **Marker** | **Color/Format** | **Clone** | **Company** |
| CD3 | R718 | OKT3 | BD Biosciences |
| CD4 | BUV395 | L200 | BD Biosciences |
| CCR6 | PE | G034E3 | Biolegend |
| CD62L | APC | [DREG-56](https://www.biolegend.com/en-us/search-results?Clone=DREG-56) | Biolegend |
| CD69 | BV605 | FN50 | BD Biosciences |
| CD38 | PE-Cy7 | HB-7 | Biolegend |
| CXCR3 | AF488 | 1C6/CXCR3 | BD Pharmigen |
| CD45RA | eFluo450 | HI100 | eBioscience (Thermo Fisher) |
| **Treg PANEL** |  |  |  |
| CD3 | R718 | OKT3 | BD Biosciences |
| CCR4 | BV605 |  | BD Biosciences |
| CD4 | BUV395 | L291H4 | Biolegend |
| CD25 | APC | L200 | BD Biosciences |
| PD-1 | BV711 | BC96 | Biolegend |
| FoxP3 | PE | EH12.2H7 | Biolegend |
| HLA-G | AF488 | PCH101 | eBioscience (Thermo Fisher) |
| CTLA-4 | BV421 | 87G | Biolegend |
| **NK PANEL** |  |  |  |
| CD16 | BUV395 | 3G8 | BD Biosciences |
| CD3 -CD14- CD20 | V450 | SP34-2 | BD Biosciences |
| CD49a | PE | TS2/7 | Biolegend |
| CD56 | BUV737 | NCAM16.2 | BD Biosciences |
| CD57 | BV711 | QA17A04 | Biolegend |
| CD69 | BV605 | FN50 | BD Biosciences |
| CD9 | APC | HI9a | Biolegend |
| NKG2A | FITC | REA110 | Miltenyi |
| NKp46 | PE-Cy7 | BAB281 | Beckman Coulter |
| **DC PANEL** |  |  |  |
| CD11c | APC | 3.9 | Biolegend |
| CD16 | BUV395 | 3G8 | BD Biosciences |
| CD3 | V450 | SP34-2 | BD Biosciences |
| CD20 | V450 | L27 | BD Biosciences |
| HLA-G | PE | 87G | Biolegend |
| HLA-DR | BV605 | L243 | Biolegend |
| CD141 | PE-Cy7 | M80 | Biolegend |
| CD163 | FITC | GHI/61 | Biolegend |
| CD86 | BV711 | IT2.2 | Biolegend |
| CD14 | BUV737 |  | Biolegend |
