# Supplementary material for: Antiretroviral Therapy at Conception Leads to Lower Peripheral CD49a+ NK Cells and Higher SERPINB2
Source: J Immunol Res. 2025 May 21;2025:4771787. doi: 10.1155/jimr/4771787 (PMC12119168; doi:10.1155/jimr/4771787)
Supplement: Supporting Information 6 — Table S6: Immune phenotyping biomarker percentages by ARV group, within different parent markers, among all women. [file 4771787.f6.docx]

**Table S6: Immune phenotyping biomarker percentages by ARV group, within different parent markers, among all women**

| **Biomarker (%)** |  | **Women on ART at Conception ¹** | **Women initiated ART at ≥ the 2nd trimester ²** | **P-Value ³** |
| --- | --- | --- | --- | --- |
| *Live Cells* | | | | |
| CD3-;CD20- | N | 29 | 27 | 0.59 |
|  | Mean (s.d.) | 10.77 (17.18) | 8.17 (7.31) |  |
|  | Median (Q1, Q3) | 5.96 (3.34, 11.70) | 5.85 (3.13, 7.92) |  |
|  | Min, Max | 1.04, 96.10 | 1.92, 34.00 |  |
| CD3-;CD20-;CD11C+ | N | 29 | 27 | 0.38 |
|  | Mean (s.d.) | 4.46 (3.29) | 3.41 (2.17) |  |
|  | Median (Q1, Q3) | 3.61 (1.79, 7.17) | 3.08 (1.43, 4.61) |  |
|  | Min, Max | 0.28, 10.90 | 0.60, 9.43 |  |
| CD3-;CD20-;CD11C+;CD14+ | N | 29 | 27 | 0.65 |
|  | Mean (s.d.) | 0.51 (0.78) | 0.37 (0.40) |  |
|  | Median (Q1, Q3) | 0.26 (0.14, 0.49) | 0.23 (0.10, 0.57) |  |
|  | Min, Max | 0.00, 3.77 | 0.00, 1.76 |  |
| CD3-;CD20-;CD11C+;CD141+ | N | 29 | 27 | 0.46 |
|  | Mean (s.d.) | 0.55 (0.90) | 0.38 (0.45) |  |
|  | Median (Q1, Q3) | 0.25 (0.15, 0.59) | 0.16 (0.11, 0.43) |  |
|  | Min, Max | 0.03, 4.27 | 0.00, 1.68 |  |
| CD3-;CD20-;CD11C+;CD86+ | N | 29 | 27 | 0.96 |
|  | Mean (s.d.) | 0.66 (0.83) | 0.63 (0.65) |  |
|  | Median (Q1, Q3) | 0.46 (0.17, 0.68) | 0.37 (0.22, 0.88) |  |
|  | Min, Max | 0.03, 3.76 | 0.00, 2.33 |  |
| CD3-;CD20-;CD11C+;DR+ | N | 29 | 27 | 0.79 |
|  | Mean (s.d.) | 1.82 (1.71) | 1.40 (1.06) |  |
|  | Median (Q1, Q3) | 1.06 (0.60, 2.77) | 1.05 (0.63, 1.71) |  |
|  | Min, Max | 0.05, 6.15 | 0.19, 4.54 |  |
| CD3-;CD20-;CD11C+;HLAG+ | N | 29 | 27 | 0.22 |
|  | Mean (s.d.) | 0.14 (0.28) | 0.13 (0.27) |  |
|  | Median (Q1, Q3) | 0.07 (0.03, 0.11) | 0.04 (0.01, 0.09) |  |
|  | Min, Max | 0.00, 1.50 | 0.00, 1.29 |  |
| CD3-;CD20-;CD11cHigh;CD163+ | N | 29 | 27 | 0.90 |
|  | Mean (s.d.) | 0.50 (0.91) | 0.37 (0.43) |  |
|  | Median (Q1, Q3) | 0.22 (0.12, 0.33) | 0.21 (0.08, 0.56) |  |
|  | Min, Max | 0.00, 4.22 | 0.00, 1.69 |  |
| CD3-;CD20-;CD14+ | N | 29 | 27 | 0.62 |
|  | Mean (s.d.) | 0.59 (0.79) | 0.46 (0.49) |  |
|  | Median (Q1, Q3) | 0.32 (0.19, 0.60) | 0.28 (0.17, 0.63) |  |
|  | Min, Max | 0.00, 3.82 | 0.00, 2.28 |  |
| CD3-;CD20-;CD14+;CD16- | N | 29 | 27 | 0.55 |
|  | Mean (s.d.) | 0.32 (0.34) | 0.30 (0.35) |  |
|  | Median (Q1, Q3) | 0.24 (0.13, 0.39) | 0.19 (0.09, 0.40) |  |
|  | Min, Max | 0.00, 1.72 | 0.00, 1.75 |  |
| CD3-;CD20-;CD14+;CD16+ | N | 29 | 27 | 0.58 |
|  | Mean (s.d.) | 0.18 (0.31) | 0.10 (0.11) |  |
|  | Median (Q1, Q3) | 0.06 (0.03, 0.24) | 0.06 (0.02, 0.16) |  |
|  | Min, Max | 0.00, 1.48 | 0.00, 0.43 |  |
| CD3-;CD20-;CD86+ | N | 29 | 27 | 0.87 |
|  | Mean (s.d.) | 1.25 (0.92) | 1.35 (1.19) |  |
|  | Median (Q1, Q3) | 1.07 (0.70, 1.66) | 1.04 (0.54, 1.76) |  |
|  | Min, Max | 0.09, 3.89 | 0.10, 5.45 |  |
| CD3-;CD20-;HLA-G+ | N | 29 | 27 | 0.47 |
|  | Mean (s.d.) | 0.34 (0.84) | 0.24 (0.42) |  |
|  | Median (Q1, Q3) | 0.13 (0.07, 0.19) | 0.12 (0.05, 0.20) |  |
|  | Min, Max | 0.00, 4.49 | 0.00, 2.10 |  |
| CD3+;CD20+;CD14+;NKG2A+ | N | 27 | 27 | 0.26 |
|  | Mean (s.d.) | 1.62 (1.23) | 1.21 (0.87) |  |
|  | Median (Q1, Q3) | 1.49 (0.64, 2.38) | 0.92 (0.57, 1.94) |  |
|  | Min, Max | 0.01, 4.61 | 0.06, 3.16 |  |
| CD3-;CD20-;CD14-;CD16-;CD56dim | N | 27 | 27 | 0.79 |
|  | Mean (s.d.) | 0.21 (0.18) | 0.20 (0.14) |  |
|  | Median (Q1, Q3) | 0.18 (0.06, 0.27) | 0.17 (0.11, 0.26) |  |
|  | Min, Max | 0.00, 0.62 | 0.00, 0.70 |  |
| CD3-;CD20-;CD14-;CD16brigh;CD56dim | N | 27 | 27 | 0.49 |
|  | Mean (s.d.) | 1.05 (1.56) | 0.65 (0.78) |  |
|  | Median (Q1, Q3) | 0.71 (0.04, 1.41) | 0.33 (0.08, 0.87) |  |
|  | Min, Max | 0.00, 7.53 | 0.00, 2.46 |  |
| CD3-;CD20-;CD14-;CD16bright;CD56- | N | 27 | 27 | 0.27 |
|  | Mean (s.d.) | 0.20 (0.38) | 0.08 (0.16) |  |
|  | Median (Q1, Q3) | 0.06 (0.01, 0.13) | 0.04 (0.01, 0.07) |  |
|  | Min, Max | 0.00, 1.60 | 0.00, 0.83 |  |
| CD3-;CD20-;CD14-;CD16dim;CD56bright | N | 27 | 27 | 0.77 |
|  | Mean (s.d.) | 0.15 (0.22) | 0.10 (0.14) |  |
|  | Median (Q1, Q3) | 0.06 (0.00, 0.23) | 0.05 (0.00, 0.16) |  |
|  | Min, Max | 0.00, 0.86 | 0.00, 0.56 |  |
| CD3-;CD20-;CD14-;CD16im;CD56dim | N | 27 | 27 | 0.40 |
|  | Mean (s.d.) | 0.39 (0.34) | 0.32 (0.34) |  |
|  | Median (Q1, Q3) | 0.33 (0.14, 0.54) | 0.23 (0.11, 0.40) |  |
|  | Min, Max | 0.00, 1.37 | 0.00, 1.68 |  |
| CD3-;CD20-;CD14-;CD56+ | N | 27 | 27 | 0.34 |
|  | Mean (s.d.) | 3.42 (2.96) | 2.36 (1.89) |  |
|  | Median (Q1, Q3) | 3.28 (1.00, 4.93) | 1.64 (1.05, 3.92) |  |
|  | Min, Max | 0.13, 12.50 | 0.22, 7.66 |  |
| CD3-;CD20-;CD14-;CD56+(All) | N | 27 | 27 | 0.52 |
|  | Mean (s.d.) | 1.99 (2.07) | 1.46 (1.33) |  |
|  | Median (Q1, Q3) | 2.02 (0.51, 2.63) | 0.85 (0.51, 2.60) |  |
|  | Min, Max | 0.03, 10.00 | 0.11, 5.01 |  |
| CD3-;CD20-;CD14-;CD56+;NKG2A+ | N | 27 | 27 | 0.48 |
|  | Mean (s.d.) | 1.19 (1.38) | 0.88 (0.80) |  |
|  | Median (Q1, Q3) | 1.08 (0.27, 1.71) | 0.73 (0.25, 1.44) |  |
|  | Min, Max | 0.00, 7.01 | 0.00, 2.99 |  |
| CD3-;CD20-;CD14-;CD56high;NKG2Ahigh | N | 27 | 27 | 0.88 |
|  | Mean (s.d.) | 0.35 (0.48) | 0.27 (0.32) |  |
|  | Median (Q1, Q3) | 0.15 (0.02, 0.45) | 0.14 (0.02, 0.47) |  |
|  | Min, Max | 0.00, 1.93 | 0.00, 1.26 |  |
| CD3-;CD20-;CD14-;NKG2A+ | N | 27 | 27 | 0.73 |
|  | Mean (s.d.) | 1.69 (1.66) | 1.40 (1.04) |  |
|  | Median (Q1, Q3) | 1.48 (0.38, 2.52) | 1.14 (0.54, 1.97) |  |
|  | Min, Max | 0.00, 8.03 | 0.13, 4.19 |  |
| DR+ | N | 29 | 27 | 1.00 |
|  | Mean (s.d.) | 18.57 (8.08) | 19.07 (9.09) |  |
|  | Median (Q1, Q3) | 18.20 (12.60, 24.20) | 17.20 (13.90, 24.70) |  |
|  | Min, Max | 3.34, 36.80 | 4.86, 44.80 |  |
| *CD3+;CD4+* | | | | |
| CD25+ | N | 26 | 28 | 0.80 |
|  | Mean (s.d.) | 28.68 (10.05) | 29.68 (11.11) |  |
|  | Median (Q1, Q3) | 28.50 (21.80, 34.90) | 29.25 (21.55, 37.55) |  |
|  | Min, Max | 11.30, 54.20 | 8.09, 51.00 |  |
| CD38+ | N | 29 | 26 | 0.39 |
|  | Mean (s.d.) | 69.33 (14.43) | 73.05 (10.77) |  |
|  | Median (Q1, Q3) | 75.10 (59.90, 78.50) | 75.70 (68.30, 80.70) |  |
|  | Min, Max | 33.10, 88.20 | 43.60, 86.70 |  |
| FOXP3+ | N | 26 | 28 | 0.62 |
|  | Mean (s.d.) | 9.28 (8.95) | 7.47 (3.42) |  |
|  | Median (Q1, Q3) | 7.51 (5.63, 9.25) | 6.64 (5.12, 9.01) |  |
|  | Min, Max | 2.99, 50.00 | 1.04, 17.40 |  |
| CXCR3-;CCR6+ | N | 29 | 26 | 0.88 |
|  | Mean (s.d.) | 9.63 (9.28) | 7.49 (5.29) |  |
|  | Median (Q1, Q3) | 6.66 (3.27, 12.00) | 6.38 (2.92, 11.80) |  |
|  | Min, Max | 0.00, 33.20 | 0.80, 21.20 |  |
| CXCR3+;CCR6- | N | 29 | 26 | 0.24 |
|  | Mean (s.d.) | 3.19 (3.78) | 3.82 (3.60) |  |
|  | Median (Q1, Q3) | 1.33 (0.56, 5.48) | 2.39 (0.84, 7.01) |  |
|  | Min, Max | 0.00, 15.80 | 0.12, 11.90 |  |
| CXCR3+;CCR6+ | N | 29 | 26 | 0.68 |
|  | Mean (s.d.) | 2.12 (1.91) | 2.08 (1.41) |  |
|  | Median (Q1, Q3) | 1.30 (0.81, 3.13) | 1.71 (0.92, 3.02) |  |
|  | Min, Max | 0.00, 6.93 | 0.45, 5.72 |  |
| PD1+ | N | 26 | 28 | 0.08 |
|  | Mean (s.d.) | 19.89 (13.38) | 13.41 (5.38) |  |
|  | Median (Q1, Q3) | 17.25 (9.83, 26.50) | 12.75 (9.06, 18.30) |  |
|  | Min, Max | 5.52, 59.20 | 4.17, 24.60 |  |
| CD69+ | N | 29 | 26 | 0.08 |
|  | Mean (s.d.) | 32.08 (22.24) | 44.17 (22.52) |  |
|  | Median (Q1, Q3) | 29.90 (9.70, 51.40) | 48.80 (34.40, 54.70) |  |
|  | Min, Max | 0.00, 70.80 | 1.23, 79.80 |  |
| CCR6+ | N | 29 | 26 | 0.88 |
|  | Mean (s.d.) | 12.31 (10.24) | 10.32 (6.44) |  |
|  | Median (Q1, Q3) | 7.76 (5.82, 16.90) | 8.62 (4.91, 13.50) |  |
|  | Min, Max | 0.00, 38.10 | 1.62, 28.40 |  |
| CD25high;FOXP3+ | N | 26 | 28 | 0.71 |
|  | Mean (s.d.) | 5.03 (4.50) | 4.06 (1.88) |  |
|  | Median (Q1, Q3) | 4.40 (2.14, 5.58) | 4.50 (3.00, 5.09) |  |
|  | Min, Max | 1.49, 25.00 | 0.00, 7.94 |  |
| CD45RA-;CD62L- | N | 29 | 26 | 0.68 |
|  | Mean (s.d.) | 6.13 (8.91) | 4.81 (3.19) |  |
|  | Median (Q1, Q3) | 4.07 (2.96, 5.78) | 3.65 (2.62, 5.63) |  |
|  | Min, Max | 0.00, 50.00 | 1.62, 13.20 |  |
| CD45RA-;CD62L+ | N | 29 | 26 | 0.09 |
|  | Mean (s.d.) | 23.60 (12.47) | 20.17 (9.71) |  |
|  | Median (Q1, Q3) | 22.90 (18.60, 32.90) | 19.15 (16.00, 22.70) |  |
|  | Min, Max | 0.00, 51.00 | 5.38, 57.00 |  |
| CD45RA+;CD62L+ | N | 29 | 26 | 0.98 |
|  | Mean (s.d.) | 27.47 (16.15) | 28.38 (17.13) |  |
|  | Median (Q1, Q3) | 28.30 (15.80, 41.90) | 30.10 (10.90, 37.70) |  |
|  | Min, Max | 0.00, 50.90 | 0.49, 59.10 |  |
| FOXP3+;CCR4+ | N | 26 | 28 | 0.67 |
|  | Mean (s.d.) | 2.72 (1.67) | 2.48 (1.42) |  |
|  | Median (Q1, Q3) | 2.68 (1.53, 3.33) | 2.31 (1.48, 3.34) |  |
|  | Min, Max | 0.00, 7.50 | 0.00, 6.19 |  |
| FOXP3+;PD1+ | N | 26 | 28 | 0.88 |
|  | Mean (s.d.) | 2.45 (4.78) | 1.60 (1.43) |  |
|  | Median (Q1, Q3) | 1.05 (0.74, 1.65) | 1.23 (0.71, 1.98) |  |
|  | Min, Max | 0.26, 25.00 | 0.00, 6.43 |  |
| CCR4+ | N | 26 | 28 | 0.19 |
|  | Mean (s.d.) | 26.06 (16.66) | 20.02 (8.10) |  |
|  | Median (Q1, Q3) | 23.10 (15.30, 32.90) | 19.30 (14.00, 24.80) |  |
|  | Min, Max | 0.00, 77.80 | 8.33, 40.90 |  |
| CD45RA- | N | 29 | 26 | 0.06 |
|  | Mean (s.d.) | 29.72 (13.70) | 24.98 (11.26) |  |
|  | Median (Q1, Q3) | 27.98 (22.49, 37.97) | 22.32 (19.42, 29.15) |  |
|  | Min, Max | 4.85, 58.10 | 7.07, 67.40 |  |
| CXCR3+ | N | 29 | 26 | 0.44 |
|  | Mean (s.d.) | 5.31 (5.21) | 5.89 (4.91) |  |
|  | Median (Q1, Q3) | 3.39 (1.31, 7.21) | 3.79 (1.84, 9.80) |  |
|  | Min, Max | 0.00, 21.22 | 0.61, 16.92 |  |
| Ratio of CCR6+CXCR3-/FOXP3+ | N | 26 | 24 | 0.83 |
|  | Mean (s.d.) | 1.49 (1.35) | 1.19 (0.74) |  |
|  | Median (Q1, Q3) | 1.10 (0.32, 2.12) | 1.09 (0.57, 1.75) |  |
|  | Min, Max | 0.00, 4.53 | 0.08, 2.81 |  |
| Ratio of CCR6+CXCR3+/FOXP3+ | N | 26 | 24 | 0.65 |
|  | Mean (s.d.) | 0.33 (0.36) | 0.41 (0.49) |  |
|  | Median (Q1, Q3) | 0.27 (0.10, 0.36) | 0.21 (0.13, 0.55) |  |
|  | Min, Max | 0.00, 1.73 | 0.04, 2.40 |  |
| Ratio of CCR6+/FOXP3+ | N | 26 | 24 | 0.96 |
|  | Mean (s.d.) | 1.84 (1.56) | 1.81 (1.63) |  |
|  | Median (Q1, Q3) | 1.46 (0.58, 2.62) | 1.45 (1.03, 2.51) |  |
|  | Min, Max | 0.00, 5.18 | 0.20, 8.41 |  |
| *CD3+* | | | | |
| CD38+ | N | 29 | 26 | 0.40 |
|  | Mean (s.d.) | 63.08 (19.24) | 66.83 (16.52) |  |
|  | Median (Q1, Q3) | 66.20 (61.20, 75.40) | 71.95 (64.00, 75.80) |  |
|  | Min, Max | 0.81, 83.30 | 5.69, 82.10 |  |
| CD69+ | N | 29 | 26 | 0.35 |
|  | Mean (s.d.) | 24.04 (20.50) | 29.51 (19.56) |  |
|  | Median (Q1, Q3) | 18.00 (4.84, 36.20) | 30.80 (12.50, 43.10) |  |
|  | Min, Max | 0.51, 72.90 | 0.41, 80.30 |  |
| CD4+ | N | 29 | 26 | 0.07 |
|  | Mean (s.d.) | 40.64 (17.12) | 48.59 (13.57) |  |
|  | Median (Q1, Q3) | 44.50 (36.10, 51.90) | 48.45 (40.70, 57.90) |  |
|  | Min, Max | 0.18, 66.50 | 2.47, 72.90 |  |
| *Lymphocytes* | | | | |
| CD3+ | N | 29 | 26 | 0.10 |
|  | Mean (s.d.) | 76.69 (22.48) | 83.86 (12.22) |  |
|  | Median (Q1, Q3) | 82.30 (75.90, 88.40) | 87.85 (78.90, 91.60) |  |
|  | Min, Max | 1.21, 95.70 | 40.80, 97.40 |  |
| *CD3-;CD20-* | | | | |
| CD14+;CD16- | N | 29 | 27 | 0.70 |
|  | Mean (s.d.) | 4.19 (4.32) | 4.02 (3.63) |  |
|  | Median (Q1, Q3) | 3.08 (1.91, 5.07) | 2.64 (1.70, 5.29) |  |
|  | Min, Max | 0.00, 23.50 | 0.00, 13.80 |  |
| CD14+;CD16+ | N | 29 | 27 | 0.74 |
|  | Mean (s.d.) | 1.90 (2.36) | 1.51 (1.61) |  |
|  | Median (Q1, Q3) | 1.02 (0.48, 2.18) | 1.06 (0.40, 2.21) |  |
|  | Min, Max | 0.00, 10.60 | 0.00, 7.33 |  |
| CD14+ | N | 29 | 27 | 0.84 |
|  | Mean (s.d.) | 6.89 (7.16) | 6.31 (5.47) |  |
|  | Median (Q1, Q3) | 5.04 (3.15, 7.72) | 4.16 (2.67, 10.30) |  |
|  | Min, Max | 0.00, 32.90 | 0.00, 23.70 |  |
| CD11c+ | N | 29 | 27 | 0.16 |
|  | Mean (s.d.) | 54.71 (21.15) | 48.70 (18.14) |  |
|  | Median (Q1, Q3) | 59.40 (35.90, 72.20) | 47.60 (39.20, 55.10) |  |
|  | Min, Max | 2.62, 82.70 | 9.95, 83.10 |  |
| CD86+ | N | 29 | 27 | 0.85 |
|  | Mean (s.d.) | 16.99 (9.63) | 18.11 (10.37) |  |
|  | Median (Q1, Q3) | 15.10 (9.62, 20.20) | 14.20 (11.20, 27.90) |  |
|  | Min, Max | 2.51, 43.60 | 3.51, 40.40 |  |
| CD11C+;CD14+ | N | 29 | 27 | 0.98 |
|  | Mean (s.d.) | 5.81 (7.23) | 5.19 (4.70) |  |
|  | Median (Q1, Q3) | 3.53 (2.32, 5.94) | 3.79 (1.75, 8.45) |  |
|  | Min, Max | 0.00, 32.40 | 0.00, 19.50 |  |
| CD11C+;CD141+ | N | 29 | 27 | 0.60 |
|  | Mean (s.d.) | 6.44 (8.56) | 5.39 (5.94) |  |
|  | Median (Q1, Q3) | 3.78 (2.51, 6.82) | 3.61 (1.57, 6.93) |  |
|  | Min, Max | 0.56, 40.20 | 0.00, 22.80 |  |
| CD11C+;CD86+ | N | 29 | 27 | 0.76 |
|  | Mean (s.d.) | 8.53 (8.47) | 8.53 (6.88) |  |
|  | Median (Q1, Q3) | 5.74 (4.55, 8.96) | 7.02 (3.67, 12.20) |  |
|  | Min, Max | 0.11, 42.70 | 0.00, 28.10 |  |
| CD11C+;DR+ | N | 29 | 27 | 0.79 |
|  | Mean (s.d.) | 22.71 (14.52) | 20.55 (10.42) |  |
|  | Median (Q1, Q3) | 21.60 (13.30, 25.60) | 20.20 (13.20, 29.50) |  |
|  | Min, Max | 0.17, 58.40 | 2.12, 42.40 |  |
| CD11C+;HLAG+ | N | 29 | 27 | 0.38 |
|  | Mean (s.d.) | 1.82 (3.26) | 1.54 (3.17) |  |
|  | Median (Q1, Q3) | 0.91 (0.48, 1.28) | 0.56 (0.21, 1.32) |  |
|  | Min, Max | 0.00, 16.90 | 0.00, 16.40 |  |
| CD11cHigh;CD163+ | N | 29 | 27 | 0.82 |
|  | Mean (s.d.) | 5.93 (8.78) | 5.44 (5.26) |  |
|  | Median (Q1, Q3) | 3.16 (2.21, 4.78) | 3.80 (1.57, 8.68) |  |
|  | Min, Max | 0.00, 41.20 | 0.00, 17.50 |  |
| HLAG+ | N | 29 | 27 | 0.35 |
|  | Mean (s.d.) | 3.37 (4.11) | 2.90 (5.13) |  |
|  | Median (Q1, Q3) | 1.72 (0.84, 4.68) | 1.84 (0.65, 2.88) |  |
|  | Min, Max | 0.00, 16.90 | 0.00, 26.80 |  |
| *CD3-;CD20-;CD11C+* | | | | |
| CD141+;CD163+ | N | 29 | 27 | 0.87 |
|  | Mean (s.d.) | 10.13 (14.48) | 9.52 (9.63) |  |
|  | Median (Q1, Q3) | 6.23 (3.23, 10.70) | 6.09 (1.61, 14.20) |  |
|  | Min, Max | 0.00, 68.40 | 0.00, 37.80 |  |
| CD16-;HLAG+ | N | 29 | 27 | 1.00 |
|  | Mean (s.d.) | 3.74 (8.27) | 2.09 (2.55) |  |
|  | Median (Q1, Q3) | 1.33 (0.41, 2.62) | 1.25 (0.53, 2.38) |  |
|  | Min, Max | 0.00, 41.70 | 0.00, 9.89 |  |
| CD16+;CD141- | N | 29 | 27 | 0.49 |
|  | Mean (s.d.) | 53.26 (24.85) | 49.29 (20.28) |  |
|  | Median (Q1, Q3) | 56.30 (43.60, 67.70) | 57.40 (30.30, 65.30) |  |
|  | Min, Max | 0.00, 87.30 | 15.60, 81.70 |  |
| CD86+;HLA-DRhigh | N | 29 | 27 | 0.31 |
|  | Mean (s.d.) | 13.20 (13.29) | 15.86 (12.49) |  |
|  | Median (Q1, Q3) | 9.66 (5.64, 13.60) | 13.60 (6.21, 22.30) |  |
|  | Min, Max | 0.00, 62.00 | 0.00, 45.70 |  |
| *CD3+;CD4-* | | | | |
| CD25+ | N | 26 | 28 | 0.36 |
|  | Mean (s.d.) | 4.95 (10.12) | 4.20 (3.58) |  |
|  | Median (Q1, Q3) | 2.58 (1.41, 4.62) | 2.83 (2.00, 5.26) |  |
|  | Min, Max | 0.60, 53.70 | 0.64, 18.30 |  |
| CD38+ | N | 29 | 26 | 0.22 |
|  | Mean (s.d.) | 67.36 (21.06) | 71.83 (18.19) |  |
|  | Median (Q1, Q3) | 68.70 (62.10, 82.30) | 74.40 (70.30, 82.70) |  |
|  | Min, Max | 0.72, 89.10 | 4.75, 87.70 |  |
| FOXP3+ | N | 26 | 28 | 0.62 |
|  | Mean (s.d.) | 0.76 (0.94) | 1.33 (2.21) |  |
|  | Median (Q1, Q3) | 0.33 (0.20, 0.98) | 0.40 (0.18, 1.31) |  |
|  | Min, Max | 0.10, 3.66 | 0.00, 9.97 |  |
| CXCR3-;CCR6+ | N | 29 | 26 | 0.35 |
|  | Mean (s.d.) | 4.79 (4.22) | 3.49 (2.35) |  |
|  | Median (Q1, Q3) | 3.32 (1.98, 5.04) | 2.97 (1.72, 3.99) |  |
|  | Min, Max | 0.88, 19.40 | 0.52, 10.20 |  |
| CXCR3+;CCR6- | N | 29 | 26 | 0.78 |
|  | Mean (s.d.) | 14.37 (16.17) | 11.02 (9.82) |  |
|  | Median (Q1, Q3) | 8.15 (0.99, 25.60) | 8.04 (1.95, 20.30) |  |
|  | Min, Max | 0.40, 52.50 | 0.12, 31.30 |  |
| CXCR3+;CCR6+ | N | 29 | 26 | 0.66 |
|  | Mean (s.d.) | 2.19 (2.30) | 1.48 (1.03) |  |
|  | Median (Q1, Q3) | 1.29 (0.63, 2.84) | 1.21 (0.74, 1.60) |  |
|  | Min, Max | 0.30, 9.92 | 0.33, 4.05 |  |
| PD1+ | N | 26 | 28 | 0.76 |
|  | Mean (s.d.) | 19.56 (6.94) | 21.24 (10.54) |  |
|  | Median (Q1, Q3) | 18.25 (14.30, 25.80) | 18.45 (13.75, 28.65) |  |
|  | Min, Max | 8.83, 33.10 | 6.54, 47.60 |  |
| CD69+ | N | 29 | 26 | 0.38 |
|  | Mean (s.d.) | 34.85 (26.24) | 41.55 (25.43) |  |
|  | Median (Q1, Q3) | 31.50 (10.10, 60.70) | 50.65 (15.40, 60.70) |  |
|  | Min, Max | 1.05, 79.70 | 0.76, 83.00 |  |
| CCR6+ | N | 29 | 26 | 0.37 |
|  | Mean (s.d.) | 6.97 (5.53) | 4.97 (2.70) |  |
|  | Median (Q1, Q3) | 4.96 (3.25, 8.26) | 4.25 (2.77, 6.95) |  |
|  | Min, Max | 1.40, 22.02 | 0.85, 11.00 |  |
| CD45RA-;CD62L- | N | 29 | 26 | 0.72 |
|  | Mean (s.d.) | 3.36 (5.66) | 3.52 (6.68) |  |
|  | Median (Q1, Q3) | 1.66 (0.93, 2.86) | 1.68 (0.95, 2.62) |  |
|  | Min, Max | 0.00, 25.40 | 0.37, 34.00 |  |
| CD45RA-;CD62L+ | N | 29 | 26 | 0.44 |
|  | Mean (s.d.) | 6.57 (8.23) | 5.82 (7.60) |  |
|  | Median (Q1, Q3) | 3.92 (2.68, 7.14) | 3.17 (2.16, 6.09) |  |
|  | Min, Max | 0.42, 42.60 | 0.40, 35.00 |  |
| CD45RA+;CD62L+ | N | 29 | 26 | 0.76 |
|  | Mean (s.d.) | 25.95 (16.03) | 25.15 (15.10) |  |
|  | Median (Q1, Q3) | 26.60 (15.00, 39.10) | 23.80 (14.70, 34.40) |  |
|  | Min, Max | 0.06, 60.80 | 0.42, 64.70 |  |
| CCR4+ | N | 26 | 28 | 0.70 |
|  | Mean (s.d.) | 4.32 (7.35) | 4.09 (4.71) |  |
|  | Median (Q1, Q3) | 2.45 (1.99, 3.36) | 2.49 (1.81, 4.77) |  |
|  | Min, Max | 0.95, 38.80 | 0.86, 24.50 |  |
| CD45RA- | N | 29 | 26 | 0.47 |
|  | Mean (s.d.) | 9.93 (11.13) | 9.34 (11.77) |  |
|  | Median (Q1, Q3) | 5.85 (4.28, 8.76) | 4.98 (3.09, 8.71) |  |
|  | Min, Max | 0.98, 45.79 | 0.80, 44.82 |  |
| CXCR3+ | N | 29 | 26 | 0.80 |
|  | Mean (s.d.) | 16.56 (17.31) | 12.50 (10.40) |  |
|  | Median (Q1, Q3) | 10.71 (1.89, 26.55) | 9.29 (2.37, 23.50) |  |
|  | Min, Max | 1.05, 55.68 | 0.45, 33.55 |  |
| Ratio of CCR6+CXCR3-/FOXP3+ | N | 26 | 24 | 0.70 |
|  | Mean (s.d.) | 18.31 (27.69) | 155,845.04 (763,421.81) |  |
|  | Median (Q1, Q3) | 7.00 (2.34, 26.36) | 7.28 (3.05, 19.04) |  |
|  | Min, Max | 0.49, 138.57 | 0.90, 3,740,000.00 |  |
| Ratio of CCR6+CXCR3+/FOXP3+ | N | 26 | 24 | 0.93 |
|  | Mean (s.d.) | 7.91 (11.65) | 58,338.17 (285,772.77) |  |
|  | Median (Q1, Q3) | 2.63 (1.05, 10.14) | 3.85 (1.24, 8.19) |  |
|  | Min, Max | 0.26, 51.01 | 0.27, 1,400,000.00 |  |
| Ratio of CCR6+/FOXP3+ | N | 26 | 24 | 0.87 |
|  | Mean (s.d.) | 26.23 (33.41) | 214,183.21 (1,049,194.58) |  |
|  | Median (Q1, Q3) | 10.22 (4.74, 37.04) | 9.59 (5.11, 27.56) |  |
|  | Min, Max | 1.13, 148.71 | 1.20, 5,140,000.00 |  |
| *CD3+;CD4-;CD45RA-;CD62L-* | | | | |
| CD38+ | N | 29 | 26 | 0.20 |
|  | Mean (s.d.) | 41.36 (21.30) | 48.41 (24.40) |  |
|  | Median (Q1, Q3) | 39.10 (26.90, 55.40) | 49.15 (36.10, 70.20) |  |
|  | Min, Max | 0.00, 80.60 | 0.00, 83.20 |  |
| CXCR3-;CCR6+ | N | 29 | 26 | 0.35 |
|  | Mean (s.d.) | 7.67 (9.32) | 12.17 (19.99) |  |
|  | Median (Q1, Q3) | 4.53 (1.34, 9.78) | 5.14 (2.27, 16.20) |  |
|  | Min, Max | 0.00, 34.20 | 0.75, 100.00 |  |
| CXCR3+;CCR6- | N | 29 | 26 | 0.77 |
|  | Mean (s.d.) | 19.97 (20.85) | 19.64 (17.49) |  |
|  | Median (Q1, Q3) | 12.50 (1.09, 37.60) | 20.70 (3.17, 30.00) |  |
|  | Min, Max | 0.00, 64.00 | 0.00, 70.00 |  |
| CXCR3+;CCR6+ | N | 29 | 26 | 0.72 |
|  | Mean (s.d.) | 4.59 (5.69) | 5.31 (7.07) |  |
|  | Median (Q1, Q3) | 3.01 (0.82, 5.56) | 2.87 (1.14, 6.09) |  |
|  | Min, Max | 0.00, 26.30 | 0.00, 30.80 |  |
| CD69+ | N | 29 | 26 | 0.26 |
|  | Mean (s.d.) | 33.62 (25.89) | 41.60 (25.38) |  |
|  | Median (Q1, Q3) | 34.80 (8.90, 57.90) | 46.15 (13.80, 58.40) |  |
|  | Min, Max | 0.00, 79.30 | 1.26, 100.00 |  |
| CCR6+ | N | 29 | 26 | 0.46 |
|  | Mean (s.d.) | 12.27 (12.56) | 17.48 (21.46) |  |
|  | Median (Q1, Q3) | 8.34 (4.35, 15.95) | 8.56 (4.68, 22.03) |  |
|  | Min, Max | 0.00, 40.40 | 1.54, 100.00 |  |
| CXCR3+ | N | 29 | 26 | 0.74 |
|  | Mean (s.d.) | 24.56 (23.57) | 24.95 (21.20) |  |
|  | Median (Q1, Q3) | 15.83 (2.35, 41.19) | 24.00 (5.13, 42.85) |  |
|  | Min, Max | 0.00, 66.66 | 0.00, 77.30 |  |
| *CD3+;CD4-;CD45RA-;CD62L+* | | | | |
| CD38+ | N | 29 | 26 | 0.10 |
|  | Mean (s.d.) | 35.52 (14.30) | 44.80 (23.32) |  |
|  | Median (Q1, Q3) | 35.00 (25.00, 45.60) | 47.20 (30.40, 55.80) |  |
|  | Min, Max | 1.59, 66.00 | 0.00, 89.90 |  |
| CXCR3-;CCR6+ | N | 29 | 26 | 0.77 |
|  | Mean (s.d.) | 4.51 (3.93) | 4.83 (4.30) |  |
|  | Median (Q1, Q3) | 3.23 (1.72, 7.08) | 3.37 (1.93, 6.52) |  |
|  | Min, Max | 0.00, 13.90 | 0.00, 16.70 |  |
| CXCR3+;CCR6- | N | 29 | 26 | 0.99 |
|  | Mean (s.d.) | 21.33 (21.22) | 19.99 (18.51) |  |
|  | Median (Q1, Q3) | 22.40 (1.14, 35.50) | 15.25 (1.27, 34.00) |  |
|  | Min, Max | 0.00, 62.40 | 0.00, 49.60 |  |
| CXCR3+;CCR6+ | N | 29 | 26 | 0.55 |
|  | Mean (s.d.) | 3.11 (3.84) | 3.84 (4.41) |  |
|  | Median (Q1, Q3) | 1.52 (0.33, 4.27) | 2.46 (0.46, 5.16) |  |
|  | Min, Max | 0.00, 13.40 | 0.00, 16.70 |  |
| CD69+ | N | 29 | 26 | 0.54 |
|  | Mean (s.d.) | 25.69 (22.70) | 29.92 (22.62) |  |
|  | Median (Q1, Q3) | 21.40 (6.28, 46.50) | 32.45 (9.10, 39.60) |  |
|  | Min, Max | 0.00, 69.50 | 0.00, 100.00 |  |
| CCR6+ | N | 29 | 26 | 0.74 |
|  | Mean (s.d.) | 7.62 (6.81) | 8.67 (8.03) |  |
|  | Median (Q1, Q3) | 5.09 (2.82, 11.80) | 6.35 (2.94, 13.67) |  |
|  | Min, Max | 0.00, 25.90 | 0.00, 33.40 |  |
| CXCR3+ | N | 29 | 26 | 0.87 |
|  | Mean (s.d.) | 24.44 (24.00) | 23.83 (20.68) |  |
|  | Median (Q1, Q3) | 23.26 (1.48, 40.69) | 16.58 (2.66, 41.30) |  |
|  | Min, Max | 0.00, 64.76 | 0.00, 57.50 |  |
| *CD3+;CD4+;CD45RA-;CD62L-* | | | | |
| CD38+ | N | 29 | 26 | 0.67 |
|  | Mean (s.d.) | 39.67 (18.52) | 44.10 (11.76) |  |
|  | Median (Q1, Q3) | 44.70 (25.60, 54.80) | 42.00 (35.40, 47.90) |  |
|  | Min, Max | 0.00, 68.70 | 29.20, 82.20 |  |
| CXCR3-;CCR6+ | N | 29 | 26 | 0.72 |
|  | Mean (s.d.) | 15.35 (16.22) | 15.51 (12.04) |  |
|  | Median (Q1, Q3) | 11.00 (4.21, 22.60) | 13.45 (2.22, 24.10) |  |
|  | Min, Max | 0.00, 66.00 | 0.00, 39.90 |  |
| CXCR3+;CCR6- | N | 29 | 26 | 0.31 |
|  | Mean (s.d.) | 6.08 (7.45) | 6.59 (5.46) |  |
|  | Median (Q1, Q3) | 2.67 (0.72, 9.20) | 5.01 (2.48, 12.50) |  |
|  | Min, Max | 0.00, 26.10 | 0.00, 16.90 |  |
| CXCR3+;CCR6+ | N | 29 | 26 | 0.06 |
|  | Mean (s.d.) | 3.38 (5.95) | 3.52 (2.36) |  |
|  | Median (Q1, Q3) | 1.55 (0.33, 3.88) | 2.84 (1.93, 4.66) |  |
|  | Min, Max | 0.00, 31.80 | 0.22, 8.82 |  |
| CD69+ | N | 29 | 26 | 0.18 |
|  | Mean (s.d.) | 27.99 (21.18) | 35.72 (19.32) |  |
|  | Median (Q1, Q3) | 24.10 (8.71, 42.60) | 38.80 (22.70, 48.70) |  |
|  | Min, Max | 0.00, 64.90 | 0.68, 71.90 |  |
| CCR6+ | N | 29 | 26 | 0.52 |
|  | Mean (s.d.) | 18.73 (18.87) | 19.03 (12.75) |  |
|  | Median (Q1, Q3) | 11.33 (5.15, 29.05) | 18.50 (4.76, 30.32) |  |
|  | Min, Max | 0.00, 69.86 | 1.18, 44.56 |  |
| CXCR3+ | N | 29 | 26 | 0.27 |
|  | Mean (s.d.) | 9.46 (10.60) | 10.12 (7.18) |  |
|  | Median (Q1, Q3) | 4.45 (1.66, 19.27) | 8.66 (3.77, 16.17) |  |
|  | Min, Max | 0.00, 38.62 | 0.74, 22.77 |  |
| *CD3+;CD4+;CD45RA-;CD62L+* | | | | |
| CD38+ | N | 29 | 26 | 0.91 |
|  | Mean (s.d.) | 45.23 (14.67) | 47.18 (12.27) |  |
|  | Median (Q1, Q3) | 48.00 (35.00, 55.90) | 44.55 (38.20, 54.60) |  |
|  | Min, Max | 0.00, 66.70 | 27.50, 85.50 |  |
| CXCR3-;CCR6+ | N | 29 | 26 | 0.27 |
|  | Mean (s.d.) | 14.65 (12.94) | 16.50 (10.51) |  |
|  | Median (Q1, Q3) | 12.30 (5.82, 19.10) | 17.75 (7.30, 24.30) |  |
|  | Min, Max | 0.00, 55.70 | 0.59, 41.20 |  |
| CXCR3+;CCR6- | N | 29 | 26 | 0.29 |
|  | Mean (s.d.) | 5.89 (7.33) | 6.97 (5.79) |  |
|  | Median (Q1, Q3) | 1.54 (0.72, 10.40) | 5.27 (1.64, 11.30) |  |
|  | Min, Max | 0.00, 28.80 | 0.00, 18.00 |  |
| CXCR3+;CCR6+ | N | 29 | 26 | 0.49 |
|  | Mean (s.d.) | 2.98 (3.46) | 2.85 (2.21) |  |
|  | Median (Q1, Q3) | 1.58 (0.71, 4.41) | 2.60 (1.04, 4.09) |  |
|  | Min, Max | 0.00, 13.50 | 0.00, 8.62 |  |
| CD69+ | N | 29 | 26 | 0.33 |
|  | Mean (s.d.) | 24.51 (18.36) | 31.36 (19.46) |  |
|  | Median (Q1, Q3) | 22.60 (8.10, 37.50) | 30.30 (19.40, 42.50) |  |
|  | Min, Max | 0.00, 58.40 | 0.72, 82.50 |  |
| CCR6+ | N | 29 | 26 | 0.39 |
|  | Mean (s.d.) | 17.63 (14.77) | 19.34 (11.62) |  |
|  | Median (Q1, Q3) | 13.88 (7.16, 26.00) | 19.99 (10.60, 27.94) |  |
|  | Min, Max | 0.00, 58.62 | 0.59, 45.34 |  |
| CXCR3+ | N | 29 | 26 | 0.43 |
|  | Mean (s.d.) | 8.88 (10.22) | 9.82 (7.58) |  |
|  | Median (Q1, Q3) | 4.15 (1.47, 15.37) | 7.92 (2.68, 16.44) |  |
|  | Min, Max | 0.00, 40.70 | 0.00, 24.62 |  |
| *CD3-;CD20-;CD14-* | | | | |
| CD56DIMCD16- | N | 27 | 27 | 0.68 |
|  | Mean (s.d.) | 7.11 (6.41) | 6.15 (3.13) |  |
|  | Median (Q1, Q3) | 5.88 (3.15, 8.17) | 6.63 (3.57, 8.57) |  |
|  | Min, Max | 0.00, 29.40 | 0.00, 10.90 |  |
| CD16-;CD56+ | N | 27 | 27 | 0.48 |
|  | Mean (s.d.) | 4.97 (6.00) | 4.50 (6.39) |  |
|  | Median (Q1, Q3) | 3.44 (0.32, 7.13) | 1.96 (0.00, 5.29) |  |
|  | Min, Max | 0.00, 22.60 | 0.00, 25.50 |  |
| CD56+ | N | 27 | 27 | 0.20 |
|  | Mean (s.d.) | 54.01 (30.84) | 42.91 (30.32) |  |
|  | Median (Q1, Q3) | 63.10 (27.40, 80.10) | 39.70 (14.40, 72.40) |  |
|  | Min, Max | 0.55, 94.60 | 0.96, 93.20 |  |
| CD56dim | N | 27 | 27 | 0.22 |
|  | Mean (s.d.) | 39.53 (24.42) | 30.89 (22.83) |  |
|  | Median (Q1, Q3) | 42.10 (22.60, 63.30) | 31.40 (8.33, 53.00) |  |
|  | Min, Max | 0.00, 81.60 | 0.00, 72.60 |  |
| CD16bright;CD56- | N | 27 | 27 | 0.21 |
|  | Mean (s.d.) | 3.69 (4.74) | 2.29 (4.84) |  |
|  | Median (Q1, Q3) | 2.12 (0.34, 4.98) | 1.24 (0.34, 2.78) |  |
|  | Min, Max | 0.00, 19.00 | 0.00, 25.80 |  |
| CD16bright;CD56dim | N | 27 | 27 | 0.34 |
|  | Mean (s.d.) | 23.82 (20.11) | 17.40 (16.33) |  |
|  | Median (Q1, Q3) | 19.30 (3.77, 41.20) | 13.80 (2.27, 26.20) |  |
|  | Min, Max | 0.00, 71.20 | 0.00, 58.00 |  |
| CD16dim;CD56bright | N | 27 | 27 | 0.92 |
|  | Mean (s.d.) | 4.65 (7.01) | 3.54 (4.28) |  |
|  | Median (Q1, Q3) | 1.57 (0.00, 8.38) | 1.90 (0.00, 5.79) |  |
|  | Min, Max | 0.00, 30.90 | 0.00, 14.90 |  |
| CD16dim;CD56dim | N | 27 | 27 | 0.39 |
|  | Mean (s.d.) | 11.88 (8.83) | 9.69 (7.77) |  |
|  | Median (Q1, Q3) | 10.90 (4.76, 15.80) | 9.30 (2.69, 16.40) |  |
|  | Min, Max | 0.00, 32.10 | 0.00, 26.50 |  |
| CD49A+;CD56+ | N | 27 | 27 | 0.19 |
|  | Mean (s.d.) | 30.37 (20.07) | 23.12 (17.13) |  |
|  | Median (Q1, Q3) | 30.30 (11.70, 49.60) | 21.90 (6.40, 36.70) |  |
|  | Min, Max | 0.33, 64.30 | 0.00, 58.70 |  |
| CD56+;NKG2A+ | N | 27 | 27 | 0.38 |
|  | Mean (s.d.) | 32.61 (21.91) | 27.29 (20.73) |  |
|  | Median (Q1, Q3) | 31.30 (15.90, 49.70) | 27.50 (6.01, 44.00) |  |
|  | Min, Max | 0.00, 66.30 | 0.00, 61.80 |  |
| CD56+;NKp46+ | N | 27 | 27 | 0.80 |
|  | Mean (s.d.) | 40.63 (31.40) | 36.88 (29.50) |  |
|  | Median (Q1, Q3) | 41.60 (6.55, 67.50) | 34.70 (6.82, 65.00) |  |
|  | Min, Max | 0.00, 91.60 | 0.00, 88.90 |  |
| CD56bright | N | 27 | 27 | 0.81 |
|  | Mean (s.d.) | 9.94 (13.17) | 8.38 (9.98) |  |
|  | Median (Q1, Q3) | 4.75 (0.63, 17.10) | 4.67 (0.03, 13.90) |  |
|  | Min, Max | 0.00, 56.80 | 0.00, 32.90 |  |
| CD56bright;CD57+ | N | 27 | 27 | 0.68 |
|  | Mean (s.d.) | 7.38 (8.66) | 6.89 (8.60) |  |
|  | Median (Q1, Q3) | 3.63 (0.45, 11.40) | 3.74 (0.05, 10.80) |  |
|  | Min, Max | 0.00, 34.50 | 0.00, 28.70 |  |
| CD56dim;CD57+ | N | 27 | 27 | 0.28 |
|  | Mean (s.d.) | 11.90 (10.51) | 8.26 (8.21) |  |
|  | Median (Q1, Q3) | 10.20 (0.87, 21.40) | 5.07 (0.76, 14.60) |  |
|  | Min, Max | 0.00, 35.50 | 0.00, 27.20 |  |
| CD56high;NKG2Ahigh | N | 27 | 27 | 0.75 |
|  | Mean (s.d.) | 10.73 (12.75) | 9.12 (10.26) |  |
|  | Median (Q1, Q3) | 7.16 (0.95, 18.10) | 5.26 (0.40, 16.00) |  |
|  | Min, Max | 0.00, 50.70 | 0.00, 35.10 |  |
| CD9+;CD56+ | N | 27 | 27 | 0.24 |
|  | Mean (s.d.) | 17.52 (13.32) | 12.79 (10.13) |  |
|  | Median (Q1, Q3) | 16.50 (8.52, 23.50) | 11.80 (2.86, 17.90) |  |
|  | Min, Max | 0.00, 51.60 | 0.00, 39.50 |  |
| *CD3-;CD20-;CD14-;CD56+* | | | | |
| CD57+ | N | 27 | 27 | 0.70 |
|  | Mean (s.d.) | 60.20 (19.72) | 58.31 (19.00) |  |
|  | Median (Q1, Q3) | 61.30 (51.50, 67.00) | 58.50 (45.30, 72.50) |  |
|  | Min, Max | 0.00, 92.30 | 0.00, 85.60 |  |
| NKG2A+ | N | 27 | 27 | 0.05 |
|  | Mean (s.d.) | 52.60 (16.91) | 62.32 (19.16) |  |
|  | Median (Q1, Q3) | 55.00 (37.20, 67.50) | 60.70 (53.70, 77.30) |  |
|  | Min, Max | 15.40, 78.20 | 5.36, 100.00 |  |
| CD16-;NKG2A+ | N | 27 | 27 | 0.23 |
|  | Mean (s.d.) | 18.72 (12.71) | 23.18 (15.32) |  |
|  | Median (Q1, Q3) | 16.40 (10.60, 24.70) | 18.80 (11.80, 38.90) |  |
|  | Min, Max | 0.00, 61.60 | 0.00, 56.30 |  |
| CD16+;NKG2A | N | 27 | 27 | 0.34 |
|  | Mean (s.d.) | 33.20 (14.46) | 37.96 (19.53) |  |
|  | Median (Q1, Q3) | 31.40 (20.10, 43.20) | 36.40 (23.40, 50.00) |  |
|  | Min, Max | 6.72, 61.50 | 2.98, 100.00 |  |
| CD16+;NKG2A- | N | 27 | 27 | 0.06 |
|  | Mean (s.d.) | 31.03 (16.30) | 23.04 (15.57) |  |
|  | Median (Q1, Q3) | 30.80 (23.30, 44.60) | 20.60 (12.50, 33.60) |  |
|  | Min, Max | 0.00, 57.10 | 0.00, 61.00 |  |
| CD49A-;NKG2A+ | N | 27 | 27 | 0.66 |
|  | Mean (s.d.) | 17.98 (15.89) | 19.09 (13.67) |  |
|  | Median (Q1, Q3) | 13.00 (5.00, 29.90) | 19.40 (6.83, 30.00) |  |
|  | Min, Max | 0.00, 53.00 | 0.00, 45.00 |  |
| CD49A+;NKG2A- | N | 27 | 27 | 0.20 |
|  | Mean (s.d.) | 20.64 (16.77) | 13.46 (9.72) |  |
|  | Median (Q1, Q3) | 15.30 (7.93, 30.80) | 11.60 (7.14, 18.60) |  |
|  | Min, Max | 0.00, 59.40 | 0.00, 45.60 |  |
| CD49A+;NKG2A+ | N | 27 | 27 | 0.19 |
|  | Mean (s.d.) | 34.82 (20.73) | 42.94 (20.32) |  |
|  | Median (Q1, Q3) | 33.30 (16.70, 53.40) | 42.10 (29.20, 60.00) |  |
|  | Min, Max | 0.00, 81.80 | 2.98, 79.70 |  |
| CD57high;NKG2Ahigh | N | 27 | 27 | 0.86 |
|  | Mean (s.d.) | 7.77 (6.72) | 7.44 (5.65) |  |
|  | Median (Q1, Q3) | 5.47 (3.67, 11.50) | 7.40 (2.56, 10.90) |  |
|  | Min, Max | 0.00, 25.10 | 0.00, 23.20 |  |
| CD9-;NKG2A+ | N | 27 | 27 | 0.15 |
|  | Mean (s.d.) | 39.90 (15.91) | 47.05 (17.88) |  |
|  | Median (Q1, Q3) | 40.30 (29.50, 50.00) | 44.50 (36.60, 60.50) |  |
|  | Min, Max | 0.00, 77.40 | 1.79, 100.00 |  |
| CD9+;NKG2A- | N | 27 | 27 | 0.10 |
|  | Mean (s.d.) | 16.19 (13.65) | 11.75 (11.26) |  |
|  | Median (Q1, Q3) | 12.60 (7.90, 21.30) | 9.21 (6.07, 12.30) |  |
|  | Min, Max | 0.00, 66.70 | 0.00, 57.70 |  |
| CD9+;NKG2A+ | N | 27 | 27 | 0.41 |
|  | Mean (s.d.) | 10.90 (7.66) | 12.55 (8.35) |  |
|  | Median (Q1, Q3) | 9.22 (4.94, 16.50) | 14.00 (4.66, 18.70) |  |
|  | Min, Max | 0.00, 31.20 | 0.00, 34.60 |  |
| *Not Applicable* | | | | |
| Lymphocytes | N | 29 | 26 | 0.70 |
|  | Mean (s.d.) | 66.85 (20.52) | 67.99 (22.60) |  |
|  | Median (Q1, Q3) | 74.50 (55.00, 82.00) | 75.95 (48.90, 83.10) |  |
|  | Min, Max | 6.73, 90.30 | 12.90, 96.50 |  |

*¹ Women who were on cART (3+ drugs) at conception or initiated cART at ≤ 3 weeks gestation AND there was no drug interruption during the whole 1st trimester AND there was no drug interruption within 6 weeks before specimen collection.*

*² Women who initiated cART at ≥ the 2nd trimester AND there was no ARV exposure at all at conception or the 1st trimester AND there was no drug interruption within 6 weeks before specimen collection.*

*³ Wilcoxon rank-sum test.*
