# Supplementary material for: Antiretroviral Therapy at Conception Leads to Lower Peripheral CD49a+ NK Cells and Higher SERPINB2
Source: J Immunol Res. 2025 May 21;2025:4771787. doi: 10.1155/jimr/4771787 (PMC12119168; doi:10.1155/jimr/4771787)
Supplement: Supporting Information 7 — Table S7: Immune phenotyping biomarker percentages by ARV group, within different parent markers, among women with VL < 400 copies/mL and CD4 count ≥350 cells/mm3. [file 4771787.f7.docx]

**Table S7: Immune phenotyping biomarker percentages by ARV group, within different parent markers, among women with VL < 400 copies/mL and CD4 count ≥ 350 cells/mm³**

| **Biomarker (%)** |  | **Women on ART at Conception ¹** | **Women initiated ART at ≥ the 2nd trimester ²** | **P-Value ³** |
| --- | --- | --- | --- | --- |
| *Live Cells* | | | | |
| CD3-;CD20- | N | 11 | 19 | 0.30 |
|  | Mean (s.d.) | 7.58 (4.07) | 6.78 (4.56) |  |
|  | Median (Q1, Q3) | 8.15 (3.29, 11.20) | 5.81 (2.74, 7.42) |  |
|  | Min, Max | 1.04, 14.00 | 1.92, 19.50 |  |
| CD3-;CD20-;CD11C+ | N | 11 | 19 | 0.46 |
|  | Mean (s.d.) | 4.67 (3.62) | 3.43 (2.33) |  |
|  | Median (Q1, Q3) | 3.61 (1.86, 8.22) | 2.58 (1.42, 4.61) |  |
|  | Min, Max | 0.28, 10.90 | 0.80, 9.43 |  |
| CD3-;CD20-;CD11C+;CD14+ | N | 11 | 19 | 0.88 |
|  | Mean (s.d.) | 0.30 (0.25) | 0.40 (0.44) |  |
|  | Median (Q1, Q3) | 0.26 (0.14, 0.47) | 0.23 (0.15, 0.57) |  |
|  | Min, Max | 0.00, 0.90 | 0.02, 1.76 |  |
| CD3-;CD20-;CD11C+;CD141+ | N | 11 | 19 | 0.75 |
|  | Mean (s.d.) | 0.32 (0.27) | 0.43 (0.52) |  |
|  | Median (Q1, Q3) | 0.23 (0.15, 0.59) | 0.16 (0.11, 0.64) |  |
|  | Min, Max | 0.03, 0.90 | 0.00, 1.68 |  |
| CD3-;CD20-;CD11C+;CD86+ | N | 11 | 19 | 0.98 |
|  | Mean (s.d.) | 0.46 (0.29) | 0.69 (0.74) |  |
|  | Median (Q1, Q3) | 0.43 (0.31, 0.55) | 0.39 (0.21, 0.90) |  |
|  | Min, Max | 0.05, 1.15 | 0.00, 2.33 |  |
| CD3-;CD20-;CD11C+;DR+ | N | 11 | 19 | 1.00 |
|  | Mean (s.d.) | 1.77 (1.78) | 1.54 (1.18) |  |
|  | Median (Q1, Q3) | 1.14 (0.67, 2.77) | 1.05 (0.56, 2.36) |  |
|  | Min, Max | 0.05, 6.15 | 0.19, 4.54 |  |
| CD3-;CD20-;CD11C+;HLAG+ | N | 11 | 19 | 0.20 |
|  | Mean (s.d.) | 0.19 (0.43) | 0.09 (0.16) |  |
|  | Median (Q1, Q3) | 0.06 (0.04, 0.09) | 0.04 (0.01, 0.09) |  |
|  | Min, Max | 0.03, 1.50 | 0.00, 0.66 |  |
| CD3-;CD20-;CD11cHigh;CD163+ | N | 11 | 19 | 0.88 |
|  | Mean (s.d.) | 0.27 (0.28) | 0.44 (0.49) |  |
|  | Median (Q1, Q3) | 0.21 (0.15, 0.29) | 0.22 (0.10, 0.71) |  |
|  | Min, Max | 0.02, 1.08 | 0.00, 1.69 |  |
| CD3-;CD20-;CD14+ | N | 11 | 19 | 0.90 |
|  | Mean (s.d.) | 0.36 (0.27) | 0.49 (0.55) |  |
|  | Median (Q1, Q3) | 0.30 (0.19, 0.54) | 0.28 (0.18, 0.63) |  |
|  | Min, Max | 0.00, 1.00 | 0.03, 2.28 |  |
| CD3-;CD20-;CD14+;CD16- | N | 11 | 19 | 0.85 |
|  | Mean (s.d.) | 0.22 (0.16) | 0.33 (0.40) |  |
|  | Median (Q1, Q3) | 0.17 (0.13, 0.25) | 0.19 (0.09, 0.42) |  |
|  | Min, Max | 0.00, 0.64 | 0.00, 1.75 |  |
| CD3-;CD20-;CD14+;CD16+ | N | 11 | 19 | 0.50 |
|  | Mean (s.d.) | 0.12 (0.10) | 0.11 (0.12) |  |
|  | Median (Q1, Q3) | 0.08 (0.03, 0.16) | 0.06 (0.02, 0.16) |  |
|  | Min, Max | 0.00, 0.31 | 0.00, 0.43 |  |
| CD3-;CD20-;CD86+ | N | 11 | 19 | 0.95 |
|  | Mean (s.d.) | 0.97 (0.47) | 1.28 (1.30) |  |
|  | Median (Q1, Q3) | 0.97 (0.70, 1.08) | 0.76 (0.54, 1.76) |  |
|  | Min, Max | 0.09, 1.72 | 0.10, 5.45 |  |
| CD3-;CD20-;HLA-G+ | N | 11 | 19 | 0.26 |
|  | Mean (s.d.) | 0.25 (0.42) | 0.17 (0.22) |  |
|  | Median (Q1, Q3) | 0.10 (0.07, 0.21) | 0.07 (0.05, 0.18) |  |
|  | Min, Max | 0.05, 1.50 | 0.00, 0.80 |  |
| CD3+;CD20+;CD14+;NKG2A+ | N | 11 | 20 | 0.29 |
|  | Mean (s.d.) | 1.87 (1.62) | 1.14 (0.96) |  |
|  | Median (Q1, Q3) | 1.49 (0.22, 3.44) | 0.69 (0.53, 1.92) |  |
|  | Min, Max | 0.01, 4.61 | 0.06, 3.16 |  |
| CD3-;CD20-;CD14-;CD16-;CD56dim | N | 11 | 20 | 0.55 |
|  | Mean (s.d.) | 0.17 (0.18) | 0.18 (0.14) |  |
|  | Median (Q1, Q3) | 0.12 (0.03, 0.23) | 0.15 (0.11, 0.21) |  |
|  | Min, Max | 0.00, 0.62 | 0.00, 0.70 |  |
| CD3-;CD20-;CD14-;CD16brigh;CD56dim | N | 11 | 20 | 1.00 |
|  | Mean (s.d.) | 1.05 (2.21) | 0.55 (0.79) |  |
|  | Median (Q1, Q3) | 0.31 (0.03, 0.89) | 0.26 (0.06, 0.50) |  |
|  | Min, Max | 0.00, 7.53 | 0.00, 2.46 |  |
| CD3-;CD20-;CD14-;CD16bright;CD56- | N | 11 | 20 | 0.21 |
|  | Mean (s.d.) | 0.10 (0.13) | 0.08 (0.18) |  |
|  | Median (Q1, Q3) | 0.06 (0.01, 0.11) | 0.04 (0.02, 0.06) |  |
|  | Min, Max | 0.00, 0.48 | 0.00, 0.83 |  |
| CD3-;CD20-;CD14-;CD16dim;CD56bright | N | 11 | 20 | 1.00 |
|  | Mean (s.d.) | 0.16 (0.31) | 0.08 (0.14) |  |
|  | Median (Q1, Q3) | 0.01 (0.00, 0.07) | 0.03 (0.00, 0.09) |  |
|  | Min, Max | 0.00, 0.86 | 0.00, 0.56 |  |
| CD3-;CD20-;CD14-;CD16im;CD56dim | N | 11 | 20 | 0.73 |
|  | Mean (s.d.) | 0.39 (0.37) | 0.30 (0.37) |  |
|  | Median (Q1, Q3) | 0.16 (0.07, 0.83) | 0.19 (0.09, 0.32) |  |
|  | Min, Max | 0.01, 0.94 | 0.00, 1.68 |  |
| CD3-;CD20-;CD14-;CD56+ | N | 11 | 20 | 0.76 |
|  | Mean (s.d.) | 3.44 (3.69) | 2.13 (2.03) |  |
|  | Median (Q1, Q3) | 3.45 (0.22, 5.13) | 1.42 (0.91, 2.44) |  |
|  | Min, Max | 0.13, 12.50 | 0.22, 7.66 |  |
| CD3-;CD20-;CD14-;CD56+(All) | N | 11 | 20 | 0.97 |
|  | Mean (s.d.) | 1.96 (2.87) | 1.28 (1.40) |  |
|  | Median (Q1, Q3) | 0.73 (0.14, 2.25) | 0.68 (0.36, 1.40) |  |
|  | Min, Max | 0.03, 10.00 | 0.11, 5.01 |  |
| CD3-;CD20-;CD14-;CD56+;NKG2A+ | N | 11 | 20 | 0.85 |
|  | Mean (s.d.) | 1.26 (2.05) | 0.75 (0.83) |  |
|  | Median (Q1, Q3) | 0.45 (0.07, 1.81) | 0.37 (0.14, 1.03) |  |
|  | Min, Max | 0.00, 7.01 | 0.00, 2.99 |  |
| CD3-;CD20-;CD14-;CD56high;NKG2Ahigh | N | 11 | 20 | 0.97 |
|  | Mean (s.d.) | 0.39 (0.66) | 0.23 (0.33) |  |
|  | Median (Q1, Q3) | 0.10 (0.00, 0.32) | 0.11 (0.01, 0.24) |  |
|  | Min, Max | 0.00, 1.93 | 0.00, 1.26 |  |
| CD3-;CD20-;CD14-;NKG2A+ | N | 11 | 20 | 1.00 |
|  | Mean (s.d.) | 1.81 (2.32) | 1.29 (1.14) |  |
|  | Median (Q1, Q3) | 1.28 (0.13, 2.60) | 0.87 (0.48, 1.79) |  |
|  | Min, Max | 0.00, 8.03 | 0.13, 4.19 |  |
| DR+ | N | 11 | 19 | 0.85 |
|  | Mean (s.d.) | 16.71 (4.89) | 18.86 (9.57) |  |
|  | Median (Q1, Q3) | 17.80 (12.60, 20.50) | 17.00 (13.90, 20.50) |  |
|  | Min, Max | 10.50, 24.80 | 4.86, 44.80 |  |
| *CD3+;CD4+* | | | | |
| CD25+ | N | 10 | 20 | 0.98 |
|  | Mean (s.d.) | 26.46 (9.00) | 27.48 (11.28) |  |
|  | Median (Q1, Q3) | 28.50 (21.00, 31.00) | 24.00 (21.00, 35.65) |  |
|  | Min, Max | 11.30, 40.90 | 8.09, 51.00 |  |
| CD38+ | N | 11 | 18 | 1.00 |
|  | Mean (s.d.) | 73.21 (9.70) | 72.77 (11.57) |  |
|  | Median (Q1, Q3) | 75.70 (63.00, 81.10) | 75.05 (68.30, 80.80) |  |
|  | Min, Max | 58.00, 88.20 | 43.60, 86.70 |  |
| FOXP3+ | N | 10 | 20 | 0.69 |
|  | Mean (s.d.) | 6.64 (2.99) | 7.28 (3.91) |  |
|  | Median (Q1, Q3) | 6.27 (4.07, 8.16) | 6.52 (4.73, 7.99) |  |
|  | Min, Max | 2.99, 12.20 | 1.04, 17.40 |  |
| CXCR3-;CCR6+ | N | 11 | 18 | 0.95 |
|  | Mean (s.d.) | 8.87 (8.69) | 7.01 (5.25) |  |
|  | Median (Q1, Q3) | 5.68 (5.24, 10.10) | 6.14 (2.92, 9.80) |  |
|  | Min, Max | 0.73, 33.20 | 0.80, 21.20 |  |
| CXCR3+;CCR6- | N | 11 | 18 | 0.24 |
|  | Mean (s.d.) | 2.11 (2.22) | 3.84 (3.94) |  |
|  | Median (Q1, Q3) | 1.33 (0.56, 5.29) | 2.17 (0.60, 7.02) |  |
|  | Min, Max | 0.16, 5.79 | 0.12, 11.90 |  |
| CXCR3+;CCR6+ | N | 11 | 18 | 0.74 |
|  | Mean (s.d.) | 1.79 (1.47) | 2.07 (1.53) |  |
|  | Median (Q1, Q3) | 1.27 (0.90, 2.26) | 1.71 (0.92, 3.02) |  |
|  | Min, Max | 0.62, 5.85 | 0.45, 5.72 |  |
| PD1+ | N | 10 | 20 | 0.74 |
|  | Mean (s.d.) | 14.35 (6.75) | 12.72 (5.27) |  |
|  | Median (Q1, Q3) | 13.05 (8.51, 17.90) | 11.70 (8.81, 16.90) |  |
|  | Min, Max | 7.54, 27.20 | 4.17, 24.60 |  |
| CD69+ | N | 11 | 18 | 0.16 |
|  | Mean (s.d.) | 31.16 (22.84) | 43.20 (21.20) |  |
|  | Median (Q1, Q3) | 29.20 (9.70, 50.70) | 48.80 (34.40, 52.90) |  |
|  | Min, Max | 1.71, 70.80 | 1.23, 77.60 |  |
| CCR6+ | N | 11 | 18 | 0.91 |
|  | Mean (s.d.) | 11.43 (9.17) | 9.81 (6.42) |  |
|  | Median (Q1, Q3) | 7.76 (5.83, 12.50) | 8.47 (4.91, 12.80) |  |
|  | Min, Max | 3.16, 34.50 | 1.62, 28.40 |  |
| CD25high;FOXP3+ | N | 10 | 20 | 0.66 |
|  | Mean (s.d.) | 4.13 (2.13) | 3.61 (1.81) |  |
|  | Median (Q1, Q3) | 4.30 (2.00, 5.39) | 3.52 (2.26, 4.83) |  |
|  | Min, Max | 1.70, 8.30 | 0.00, 6.37 |  |
| CD45RA-;CD62L- | N | 11 | 18 | 0.61 |
|  | Mean (s.d.) | 4.50 (4.05) | 5.22 (3.70) |  |
|  | Median (Q1, Q3) | 3.19 (2.59, 4.08) | 3.65 (2.46, 7.25) |  |
|  | Min, Max | 1.82, 16.30 | 1.62, 13.20 |  |
| CD45RA-;CD62L+ | N | 11 | 18 | 0.10 |
|  | Mean (s.d.) | 23.22 (11.73) | 19.36 (10.83) |  |
|  | Median (Q1, Q3) | 22.90 (19.30, 33.40) | 18.35 (14.10, 21.60) |  |
|  | Min, Max | 3.69, 40.80 | 5.38, 57.00 |  |
| CD45RA+;CD62L+ | N | 11 | 18 | 0.57 |
|  | Mean (s.d.) | 32.03 (18.22) | 27.96 (19.69) |  |
|  | Median (Q1, Q3) | 38.30 (16.60, 48.70) | 30.10 (8.94, 44.20) |  |
|  | Min, Max | 0.32, 50.90 | 0.49, 59.10 |  |
| FOXP3+;CCR4+ | N | 10 | 20 | 0.76 |
|  | Mean (s.d.) | 2.07 (0.73) | 2.11 (1.46) |  |
|  | Median (Q1, Q3) | 2.01 (1.53, 2.39) | 1.80 (1.30, 2.54) |  |
|  | Min, Max | 1.18, 3.27 | 0.00, 6.19 |  |
| FOXP3+;PD1+ | N | 10 | 20 | 0.81 |
|  | Mean (s.d.) | 1.04 (0.43) | 1.65 (1.64) |  |
|  | Median (Q1, Q3) | 1.02 (0.61, 1.48) | 0.90 (0.63, 2.12) |  |
|  | Min, Max | 0.57, 1.66 | 0.00, 6.43 |  |
| CCR4+ | N | 10 | 20 | 0.64 |
|  | Mean (s.d.) | 20.05 (9.28) | 18.24 (6.88) |  |
|  | Median (Q1, Q3) | 20.25 (14.40, 27.30) | 17.90 (12.75, 21.90) |  |
|  | Min, Max | 6.51, 36.80 | 8.83, 34.70 |  |
| CD45RA- | N | 11 | 18 | 0.25 |
|  | Mean (s.d.) | 27.71 (14.58) | 24.58 (12.80) |  |
|  | Median (Q1, Q3) | 25.41 (22.49, 36.73) | 22.03 (17.83, 28.77) |  |
|  | Min, Max | 5.51, 57.10 | 7.07, 67.40 |  |
| CXCR3+ | N | 11 | 18 | 0.50 |
|  | Mean (s.d.) | 3.89 (2.50) | 5.91 (5.37) |  |
|  | Median (Q1, Q3) | 2.58 (1.87, 6.83) | 3.26 (1.76, 9.80) |  |
|  | Min, Max | 1.06, 7.21 | 0.61, 16.92 |  |
| Ratio of CCR6+CXCR3-/FOXP3+ | N | 10 | 17 | 0.62 |
|  | Mean (s.d.) | 1.43 (1.11) | 1.14 (0.74) |  |
|  | Median (Q1, Q3) | 1.19 (0.88, 1.97) | 0.98 (0.76, 1.51) |  |
|  | Min, Max | 0.06, 4.07 | 0.08, 2.81 |  |
| Ratio of CCR6+CXCR3+/FOXP3+ | N | 10 | 17 | 0.71 |
|  | Mean (s.d.) | 0.27 (0.21) | 0.44 (0.57) |  |
|  | Median (Q1, Q3) | 0.24 (0.13, 0.33) | 0.22 (0.12, 0.50) |  |
|  | Min, Max | 0.05, 0.78 | 0.04, 2.40 |  |
| Ratio of CCR6+/FOXP3+ | N | 10 | 17 | 0.63 |
|  | Mean (s.d.) | 1.75 (1.14) | 1.83 (1.86) |  |
|  | Median (Q1, Q3) | 1.53 (1.13, 2.28) | 1.43 (1.07, 1.82) |  |
|  | Min, Max | 0.26, 4.23 | 0.20, 8.41 |  |
| *CD3+* | | | | |
| CD38+ | N | 11 | 18 | 0.47 |
|  | Mean (s.d.) | 61.91 (20.33) | 65.12 (19.36) |  |
|  | Median (Q1, Q3) | 62.10 (57.60, 73.20) | 72.60 (61.40, 77.60) |  |
|  | Min, Max | 7.53, 83.30 | 5.69, 82.10 |  |
| CD69+ | N | 11 | 18 | 0.36 |
|  | Mean (s.d.) | 21.03 (20.19) | 26.95 (17.06) |  |
|  | Median (Q1, Q3) | 17.10 (4.84, 32.30) | 30.15 (12.50, 37.90) |  |
|  | Min, Max | 0.51, 61.90 | 0.41, 58.10 |  |
| CD4+ | N | 11 | 18 | 0.64 |
|  | Mean (s.d.) | 43.39 (17.19) | 47.13 (14.78) |  |
|  | Median (Q1, Q3) | 45.40 (37.10, 56.00) | 48.45 (38.90, 57.90) |  |
|  | Min, Max | 4.34, 66.50 | 2.47, 65.00 |  |
| *Lymphocytes* | | | | |
| CD3+ | N | 11 | 18 | 0.04 |
|  | Mean (s.d.) | 75.60 (25.09) | 84.42 (13.20) |  |
|  | Median (Q1, Q3) | 82.30 (78.50, 87.20) | 88.65 (80.70, 91.60) |  |
|  | Min, Max | 1.21, 90.30 | 40.80, 96.10 |  |
| *CD3-;CD20-* | | | | |
| CD14+;CD16- | N | 11 | 19 | 0.53 |
|  | Mean (s.d.) | 3.17 (2.49) | 4.63 (4.04) |  |
|  | Median (Q1, Q3) | 2.57 (1.24, 4.08) | 3.33 (1.70, 7.20) |  |
|  | Min, Max | 0.00, 7.83 | 0.00, 13.80 |  |
| CD14+;CD16+ | N | 11 | 19 | 0.81 |
|  | Mean (s.d.) | 1.36 (0.92) | 1.61 (1.79) |  |
|  | Median (Q1, Q3) | 1.24 (0.49, 2.18) | 1.06 (0.40, 2.21) |  |
|  | Min, Max | 0.00, 3.02 | 0.00, 7.33 |  |
| CD14+ | N | 11 | 19 | 0.37 |
|  | Mean (s.d.) | 4.77 (3.55) | 7.10 (5.96) |  |
|  | Median (Q1, Q3) | 3.86 (2.95, 6.54) | 4.67 (2.82, 11.40) |  |
|  | Min, Max | 0.00, 12.30 | 1.19, 23.70 |  |
| CD11c+ | N | 11 | 19 | 0.55 |
|  | Mean (s.d.) | 56.35 (23.66) | 51.73 (16.29) |  |
|  | Median (Q1, Q3) | 62.00 (31.60, 77.50) | 49.10 (41.70, 57.30) |  |
|  | Min, Max | 19.50, 82.70 | 16.20, 83.10 |  |
| CD86+ | N | 11 | 19 | 0.45 |
|  | Mean (s.d.) | 15.54 (9.47) | 18.15 (10.17) |  |
|  | Median (Q1, Q3) | 14.20 (9.09, 21.10) | 14.20 (11.70, 27.90) |  |
|  | Min, Max | 3.72, 33.50 | 3.51, 40.40 |  |
| CD11C+;CD14+ | N | 11 | 19 | 0.32 |
|  | Mean (s.d.) | 3.81 (3.25) | 5.94 (5.15) |  |
|  | Median (Q1, Q3) | 2.65 (1.80, 5.54) | 4.00 (2.19, 9.02) |  |
|  | Min, Max | 0.00, 11.10 | 0.70, 19.50 |  |
| CD11C+;CD141+ | N | 11 | 19 | 1.00 |
|  | Mean (s.d.) | 4.55 (3.18) | 6.17 (6.82) |  |
|  | Median (Q1, Q3) | 3.81 (2.22, 7.05) | 3.61 (1.57, 7.04) |  |
|  | Min, Max | 1.02, 11.00 | 0.00, 22.80 |  |
| CD11C+;CD86+ | N | 11 | 19 | 0.49 |
|  | Mean (s.d.) | 6.83 (4.35) | 9.56 (7.76) |  |
|  | Median (Q1, Q3) | 5.10 (4.50, 7.72) | 7.39 (2.90, 15.40) |  |
|  | Min, Max | 2.54, 16.00 | 0.00, 28.10 |  |
| CD11C+;DR+ | N | 11 | 19 | 0.70 |
|  | Mean (s.d.) | 21.64 (14.54) | 23.00 (10.29) |  |
|  | Median (Q1, Q3) | 21.60 (6.70, 31.40) | 22.20 (15.80, 30.10) |  |
|  | Min, Max | 4.55, 52.70 | 6.47, 42.40 |  |
| CD11C+;HLAG+ | N | 11 | 19 | 0.29 |
|  | Mean (s.d.) | 2.97 (5.00) | 1.10 (1.28) |  |
|  | Median (Q1, Q3) | 1.02 (0.43, 2.83) | 0.56 (0.21, 1.40) |  |
|  | Min, Max | 0.23, 16.90 | 0.00, 4.39 |  |
| CD11cHigh;CD163+ | N | 11 | 19 | 0.41 |
|  | Mean (s.d.) | 3.83 (3.46) | 6.30 (5.82) |  |
|  | Median (Q1, Q3) | 2.62 (2.08, 3.69) | 4.02 (1.57, 9.22) |  |
|  | Min, Max | 1.05, 13.20 | 0.00, 17.50 |  |
| HLAG+ | N | 11 | 19 | 0.52 |
|  | Mean (s.d.) | 4.69 (6.06) | 2.18 (2.06) |  |
|  | Median (Q1, Q3) | 2.52 (0.66, 6.48) | 1.84 (0.82, 2.88) |  |
|  | Min, Max | 0.36, 16.90 | 0.00, 8.74 |  |
| *CD3-;CD20-;CD11C+* | | | | |
| CD141+;CD163+ | N | 11 | 19 | 0.41 |
|  | Mean (s.d.) | 6.79 (8.55) | 10.62 (10.85) |  |
|  | Median (Q1, Q3) | 3.98 (1.89, 8.33) | 7.58 (1.61, 14.50) |  |
|  | Min, Max | 0.63, 30.60 | 0.00, 37.80 |  |
| CD16-;HLAG+ | N | 11 | 19 | 0.80 |
|  | Mean (s.d.) | 5.41 (12.17) | 1.88 (2.28) |  |
|  | Median (Q1, Q3) | 1.17 (0.30, 4.12) | 0.91 (0.53, 2.38) |  |
|  | Min, Max | 0.00, 41.70 | 0.00, 9.55 |  |
| CD16+;CD141- | N | 11 | 19 | 0.46 |
|  | Mean (s.d.) | 55.91 (25.05) | 49.78 (20.91) |  |
|  | Median (Q1, Q3) | 56.30 (38.70, 79.40) | 57.40 (30.30, 65.30) |  |
|  | Min, Max | 0.00, 84.90 | 15.60, 81.70 |  |
| CD86+;HLA-DRhigh | N | 11 | 19 | 0.40 |
|  | Mean (s.d.) | 11.56 (11.69) | 17.00 (14.21) |  |
|  | Median (Q1, Q3) | 7.37 (3.68, 16.10) | 13.60 (6.21, 31.90) |  |
|  | Min, Max | 0.00, 39.20 | 0.00, 45.70 |  |
| *CD3+;CD4-* | | | | |
| CD25+ | N | 10 | 20 | 0.76 |
|  | Mean (s.d.) | 3.40 (2.42) | 3.45 (2.08) |  |
|  | Median (Q1, Q3) | 2.50 (1.41, 4.80) | 2.83 (2.00, 4.47) |  |
|  | Min, Max | 1.26, 7.99 | 0.64, 7.76 |  |
| CD38+ | N | 11 | 18 | 0.33 |
|  | Mean (s.d.) | 65.46 (22.39) | 69.95 (21.20) |  |
|  | Median (Q1, Q3) | 65.50 (59.20, 85.40) | 72.15 (68.90, 82.90) |  |
|  | Min, Max | 6.89, 89.10 | 4.75, 87.70 |  |
| FOXP3+ | N | 10 | 20 | 0.86 |
|  | Mean (s.d.) | 1.06 (1.23) | 1.29 (2.32) |  |
|  | Median (Q1, Q3) | 0.34 (0.16, 2.09) | 0.42 (0.18, 1.31) |  |
|  | Min, Max | 0.12, 3.66 | 0.13, 9.97 |  |
| CXCR3-;CCR6+ | N | 11 | 18 | 0.15 |
|  | Mean (s.d.) | 4.86 (3.43) | 3.18 (2.39) |  |
|  | Median (Q1, Q3) | 3.90 (1.97, 6.82) | 2.71 (1.63, 3.17) |  |
|  | Min, Max | 1.03, 12.20 | 0.52, 10.20 |  |
| CXCR3+;CCR6- | N | 11 | 18 | 0.57 |
|  | Mean (s.d.) | 14.84 (16.64) | 9.84 (9.27) |  |
|  | Median (Q1, Q3) | 9.23 (0.92, 23.70) | 7.01 (1.95, 13.80) |  |
|  | Min, Max | 0.40, 52.50 | 0.12, 30.00 |  |
| CXCR3+;CCR6+ | N | 11 | 18 | 0.28 |
|  | Mean (s.d.) | 2.09 (1.54) | 1.44 (1.01) |  |
|  | Median (Q1, Q3) | 1.60 (0.69, 2.86) | 1.20 (0.74, 1.60) |  |
|  | Min, Max | 0.60, 5.79 | 0.33, 3.83 |  |
| PD1+ | N | 10 | 20 | 0.86 |
|  | Mean (s.d.) | 17.44 (5.80) | 18.73 (9.12) |  |
|  | Median (Q1, Q3) | 17.15 (13.70, 20.60) | 17.60 (10.02, 25.45) |  |
|  | Min, Max | 8.83, 29.00 | 6.54, 35.90 |  |
| CD69+ | N | 11 | 18 | 0.62 |
|  | Mean (s.d.) | 30.98 (27.10) | 38.10 (23.89) |  |
|  | Median (Q1, Q3) | 21.80 (8.74, 60.70) | 47.05 (15.40, 59.70) |  |
|  | Min, Max | 1.63, 79.70 | 0.76, 73.60 |  |
| CCR6+ | N | 11 | 18 | 0.10 |
|  | Mean (s.d.) | 6.96 (3.89) | 4.62 (2.76) |  |
|  | Median (Q1, Q3) | 7.13 (3.61, 9.69) | 3.84 (2.77, 5.47) |  |
|  | Min, Max | 2.49, 14.98 | 0.85, 11.00 |  |
| CD45RA-;CD62L- | N | 11 | 18 | 0.91 |
|  | Mean (s.d.) | 3.69 (5.97) | 4.31 (7.95) |  |
|  | Median (Q1, Q3) | 1.90 (0.77, 3.69) | 1.52 (0.92, 2.88) |  |
|  | Min, Max | 0.46, 21.30 | 0.37, 34.00 |  |
| CD45RA-;CD62L+ | N | 11 | 18 | 0.70 |
|  | Mean (s.d.) | 5.01 (3.91) | 5.94 (8.27) |  |
|  | Median (Q1, Q3) | 3.60 (1.81, 9.52) | 3.05 (1.59, 6.09) |  |
|  | Min, Max | 0.52, 12.00 | 0.45, 35.00 |  |
| CD45RA+;CD62L+ | N | 11 | 18 | 0.80 |
|  | Mean (s.d.) | 28.20 (19.81) | 26.00 (17.32) |  |
|  | Median (Q1, Q3) | 26.60 (11.20, 47.00) | 26.25 (11.40, 34.60) |  |
|  | Min, Max | 0.06, 60.80 | 0.42, 64.70 |  |
| CCR4+ | N | 10 | 20 | 0.77 |
|  | Mean (s.d.) | 2.88 (1.64) | 3.44 (2.91) |  |
|  | Median (Q1, Q3) | 2.47 (2.11, 3.49) | 2.30 (1.57, 4.92) |  |
|  | Min, Max | 0.95, 6.85 | 0.86, 12.80 |  |
| CD45RA- | N | 11 | 18 | 0.75 |
|  | Mean (s.d.) | 8.70 (9.19) | 10.25 (13.53) |  |
|  | Median (Q1, Q3) | 6.29 (2.51, 13.21) | 4.72 (2.51, 8.71) |  |
|  | Min, Max | 0.98, 33.30 | 0.82, 44.82 |  |
| CXCR3+ | N | 11 | 18 | 0.59 |
|  | Mean (s.d.) | 16.94 (17.41) | 11.28 (9.96) |  |
|  | Median (Q1, Q3) | 12.01 (2.38, 25.98) | 8.29 (2.37, 17.63) |  |
|  | Min, Max | 1.08, 55.68 | 0.45, 32.88 |  |
| Ratio of CCR6+CXCR3-/FOXP3+ | N | 10 | 17 | 0.38 |
|  | Mean (s.d.) | 17.07 (15.36) | 9.09 (7.54) |  |
|  | Median (Q1, Q3) | 18.19 (1.60, 30.50) | 7.59 (2.79, 15.29) |  |
|  | Min, Max | 0.49, 40.42 | 0.90, 22.16 |  |
| Ratio of CCR6+CXCR3+/FOXP3+ | N | 10 | 17 | 0.67 |
|  | Mean (s.d.) | 6.63 (7.19) | 5.11 (6.72) |  |
|  | Median (Q1, Q3) | 4.08 (0.70, 11.78) | 2.56 (1.23, 4.83) |  |
|  | Min, Max | 0.26, 19.00 | 0.27, 27.36 |  |
| Ratio of CCR6+/FOXP3+ | N | 10 | 17 | 0.44 |
|  | Mean (s.d.) | 23.70 (20.41) | 14.21 (12.22) |  |
|  | Median (Q1, Q3) | 30.14 (2.65, 37.04) | 9.03 (4.55, 23.81) |  |
|  | Min, Max | 1.13, 59.42 | 1.37, 39.07 |  |
| *CD3+;CD4-;CD45RA-;CD62L-* | | | | |
| CD38+ | N | 11 | 18 | 0.18 |
|  | Mean (s.d.) | 38.48 (20.77) | 48.65 (24.04) |  |
|  | Median (Q1, Q3) | 34.20 (25.50, 52.70) | 48.60 (36.30, 70.20) |  |
|  | Min, Max | 5.09, 80.60 | 1.03, 83.20 |  |
| CXCR3-;CCR6+ | N | 11 | 18 | 0.77 |
|  | Mean (s.d.) | 11.80 (13.01) | 9.32 (9.98) |  |
|  | Median (Q1, Q3) | 9.78 (0.61, 26.90) | 5.14 (2.64, 16.20) |  |
|  | Min, Max | 0.00, 34.20 | 0.75, 40.00 |  |
| CXCR3+;CCR6- | N | 11 | 18 | 0.86 |
|  | Mean (s.d.) | 21.50 (23.76) | 19.23 (17.94) |  |
|  | Median (Q1, Q3) | 12.50 (1.09, 41.70) | 18.35 (3.45, 27.30) |  |
|  | Min, Max | 0.00, 64.00 | 0.00, 70.00 |  |
| CXCR3+;CCR6+ | N | 11 | 18 | 0.67 |
|  | Mean (s.d.) | 4.36 (4.19) | 6.46 (8.17) |  |
|  | Median (Q1, Q3) | 3.33 (0.91, 5.56) | 2.87 (1.72, 7.30) |  |
|  | Min, Max | 0.00, 13.50 | 0.26, 30.80 |  |
| CD69+ | N | 11 | 18 | 0.84 |
|  | Mean (s.d.) | 35.86 (27.68) | 38.16 (23.85) |  |
|  | Median (Q1, Q3) | 38.30 (6.67, 68.00) | 44.80 (11.20, 55.30) |  |
|  | Min, Max | 1.56, 76.30 | 1.26, 70.00 |  |
| CCR6+ | N | 11 | 18 | 0.98 |
|  | Mean (s.d.) | 16.16 (15.84) | 15.78 (15.19) |  |
|  | Median (Q1, Q3) | 9.78 (2.33, 37.53) | 9.47 (4.68, 22.03) |  |
|  | Min, Max | 0.00, 40.40 | 1.54, 50.00 |  |
| CXCR3+ | N | 11 | 18 | 0.87 |
|  | Mean (s.d.) | 25.86 (24.30) | 25.69 (22.48) |  |
|  | Median (Q1, Q3) | 15.83 (2.33, 47.26) | 22.77 (5.17, 45.19) |  |
|  | Min, Max | 1.09, 66.66 | 0.38, 77.30 |  |
| *CD3+;CD4-;CD45RA-;CD62L+* | | | | |
| CD38+ | N | 11 | 18 | 0.04 |
|  | Mean (s.d.) | 31.75 (13.95) | 47.11 (23.50) |  |
|  | Median (Q1, Q3) | 28.20 (21.60, 48.40) | 49.70 (30.40, 68.20) |  |
|  | Min, Max | 15.00, 54.90 | 1.42, 89.90 |  |
| CXCR3-;CCR6+ | N | 11 | 18 | 0.57 |
|  | Mean (s.d.) | 6.37 (4.38) | 5.67 (4.67) |  |
|  | Median (Q1, Q3) | 6.83 (2.13, 7.69) | 3.84 (2.71, 8.63) |  |
|  | Min, Max | 0.90, 13.90 | 0.00, 16.70 |  |
| CXCR3+;CCR6- | N | 11 | 18 | 0.67 |
|  | Mean (s.d.) | 22.89 (20.83) | 18.83 (17.44) |  |
|  | Median (Q1, Q3) | 23.60 (0.66, 35.70) | 15.25 (1.68, 30.00) |  |
|  | Min, Max | 0.00, 59.80 | 0.00, 49.60 |  |
| CXCR3+;CCR6+ | N | 11 | 18 | 0.82 |
|  | Mean (s.d.) | 3.43 (3.47) | 4.53 (5.01) |  |
|  | Median (Q1, Q3) | 2.38 (0.56, 5.00) | 2.46 (0.66, 6.55) |  |
|  | Min, Max | 0.00, 12.00 | 0.00, 16.70 |  |
| CD69+ | N | 11 | 18 | 0.82 |
|  | Mean (s.d.) | 23.80 (22.87) | 26.12 (18.98) |  |
|  | Median (Q1, Q3) | 14.50 (2.66, 51.70) | 29.10 (8.40, 39.60) |  |
|  | Min, Max | 0.00, 59.80 | 0.00, 55.20 |  |
| CCR6+ | N | 11 | 18 | 0.98 |
|  | Mean (s.d.) | 9.80 (7.27) | 10.20 (8.87) |  |
|  | Median (Q1, Q3) | 9.63 (3.19, 12.92) | 8.73 (3.48, 16.81) |  |
|  | Min, Max | 0.90, 25.90 | 0.00, 33.40 |  |
| CXCR3+ | N | 11 | 18 | 0.82 |
|  | Mean (s.d.) | 26.32 (23.18) | 23.36 (19.85) |  |
|  | Median (Q1, Q3) | 27.15 (1.15, 47.50) | 16.58 (2.96, 39.01) |  |
|  | Min, Max | 0.00, 64.07 | 0.00, 57.50 |  |
| *CD3+;CD4+;CD45RA-;CD62L-* | | | | |
| CD38+ | N | 11 | 18 | 0.84 |
|  | Mean (s.d.) | 44.83 (16.25) | 44.11 (12.67) |  |
|  | Median (Q1, Q3) | 44.70 (25.40, 57.60) | 42.50 (35.30, 46.30) |  |
|  | Min, Max | 22.70, 68.70 | 29.20, 82.20 |  |
| CXCR3-;CCR6+ | N | 11 | 18 | 0.87 |
|  | Mean (s.d.) | 16.44 (18.15) | 14.51 (11.77) |  |
|  | Median (Q1, Q3) | 12.70 (5.08, 20.00) | 12.90 (2.22, 23.80) |  |
|  | Min, Max | 1.94, 66.00 | 0.00, 39.90 |  |
| CXCR3+;CCR6- | N | 11 | 18 | 0.36 |
|  | Mean (s.d.) | 5.42 (7.85) | 6.16 (5.45) |  |
|  | Median (Q1, Q3) | 2.67 (0.72, 6.82) | 4.05 (2.83, 8.32) |  |
|  | Min, Max | 0.00, 26.10 | 0.00, 16.90 |  |
| CXCR3+;CCR6+ | N | 11 | 18 | 0.16 |
|  | Mean (s.d.) | 4.63 (9.20) | 3.60 (2.44) |  |
|  | Median (Q1, Q3) | 1.66 (0.00, 3.86) | 2.80 (1.96, 5.62) |  |
|  | Min, Max | 0.00, 31.80 | 0.22, 8.82 |  |
| CD69+ | N | 11 | 18 | 0.38 |
|  | Mean (s.d.) | 26.98 (20.53) | 33.34 (17.88) |  |
|  | Median (Q1, Q3) | 24.10 (8.71, 41.70) | 36.90 (22.70, 47.20) |  |
|  | Min, Max | 0.00, 62.00 | 0.68, 57.60 |  |
| CCR6+ | N | 11 | 18 | 0.95 |
|  | Mean (s.d.) | 21.07 (22.27) | 18.11 (12.43) |  |
|  | Median (Q1, Q3) | 14.25 (5.15, 26.07) | 16.39 (4.76, 29.96) |  |
|  | Min, Max | 3.60, 69.86 | 1.18, 44.56 |  |
| CXCR3+ | N | 11 | 18 | 0.43 |
|  | Mean (s.d.) | 10.06 (12.43) | 9.76 (7.27) |  |
|  | Median (Q1, Q3) | 4.45 (2.77, 19.27) | 7.93 (3.77, 16.17) |  |
|  | Min, Max | 0.00, 38.62 | 0.74, 22.52 |  |
| *CD3+;CD4+;CD45RA-;CD62L+* | | | | |
| CD38+ | N | 11 | 18 | 0.98 |
|  | Mean (s.d.) | 46.39 (12.86) | 48.01 (12.59) |  |
|  | Median (Q1, Q3) | 46.70 (35.00, 56.30) | 46.05 (38.50, 54.60) |  |
|  | Min, Max | 25.10, 65.70 | 33.80, 85.50 |  |
| CXCR3-;CCR6+ | N | 11 | 18 | 0.37 |
|  | Mean (s.d.) | 15.56 (15.19) | 15.57 (9.18) |  |
|  | Median (Q1, Q3) | 13.70 (5.86, 18.00) | 17.20 (10.60, 20.40) |  |
|  | Min, Max | 0.51, 55.70 | 0.59, 32.90 |  |
| CXCR3+;CCR6- | N | 11 | 18 | 0.23 |
|  | Mean (s.d.) | 4.16 (5.02) | 6.89 (5.98) |  |
|  | Median (Q1, Q3) | 1.37 (0.41, 8.89) | 4.50 (1.64, 11.30) |  |
|  | Min, Max | 0.17, 13.50 | 0.00, 18.00 |  |
| CXCR3+;CCR6+ | N | 11 | 18 | 0.51 |
|  | Mean (s.d.) | 2.66 (3.70) | 2.77 (2.45) |  |
|  | Median (Q1, Q3) | 1.67 (0.95, 2.92) | 2.42 (1.04, 4.09) |  |
|  | Min, Max | 0.17, 13.50 | 0.00, 8.62 |  |
| CD69+ | N | 11 | 18 | 0.36 |
|  | Mean (s.d.) | 21.42 (17.98) | 28.43 (16.67) |  |
|  | Median (Q1, Q3) | 13.20 (5.23, 37.20) | 28.15 (19.40, 34.30) |  |
|  | Min, Max | 0.66, 49.20 | 0.72, 62.70 |  |
| CCR6+ | N | 11 | 18 | 0.43 |
|  | Mean (s.d.) | 18.22 (16.99) | 18.34 (10.22) |  |
|  | Median (Q1, Q3) | 15.58 (7.16, 19.67) | 18.96 (12.56, 25.42) |  |
|  | Min, Max | 0.68, 58.62 | 0.59, 34.32 |  |
| CXCR3+ | N | 11 | 18 | 0.36 |
|  | Mean (s.d.) | 6.82 (8.02) | 9.66 (8.08) |  |
|  | Median (Q1, Q3) | 3.12 (2.20, 10.37) | 6.95 (2.68, 15.19) |  |
|  | Min, Max | 0.34, 27.00 | 0.00, 24.62 |  |
| *CD3-;CD20-;CD14-* | | | | |
| CD56^dim^CD16- | N | 11 | 20 | 0.22 |
|  | Mean (s.d.) | 5.98 (6.21) | 6.38 (3.27) |  |
|  | Median (Q1, Q3) | 5.88 (0.23, 6.49) | 7.06 (3.74, 8.75) |  |
|  | Min, Max | 0.00, 18.90 | 0.00, 10.90 |  |
| CD16-;CD56+ | N | 11 | 20 | 0.72 |
|  | Mean (s.d.) | 4.56 (6.61) | 3.80 (6.33) |  |
|  | Median (Q1, Q3) | 3.80 (0.00, 6.63) | 1.53 (0.00, 4.51) |  |
|  | Min, Max | 0.00, 22.60 | 0.00, 25.50 |  |
| CD56+ | N | 11 | 20 | 0.58 |
|  | Mean (s.d.) | 47.66 (33.84) | 38.89 (29.35) |  |
|  | Median (Q1, Q3) | 50.30 (3.45, 81.10) | 34.05 (11.37, 64.55) |  |
|  | Min, Max | 0.55, 94.60 | 0.96, 93.20 |  |
| CD56dim | N | 11 | 20 | 0.73 |
|  | Mean (s.d.) | 33.41 (27.49) | 28.12 (22.64) |  |
|  | Median (Q1, Q3) | 36.30 (0.65, 47.20) | 28.40 (7.26, 42.65) |  |
|  | Min, Max | 0.00, 81.60 | 0.00, 72.60 |  |
| CD16bright;CD56- | N | 11 | 20 | 0.55 |
|  | Mean (s.d.) | 2.77 (3.34) | 2.63 (5.58) |  |
|  | Median (Q1, Q3) | 1.57 (0.65, 3.77) | 1.34 (0.60, 2.26) |  |
|  | Min, Max | 0.00, 11.50 | 0.00, 25.80 |  |
| CD16bright;CD56dim | N | 11 | 20 | 0.87 |
|  | Mean (s.d.) | 19.47 (22.44) | 15.53 (16.59) |  |
|  | Median (Q1, Q3) | 11.30 (0.20, 34.40) | 12.10 (2.06, 21.35) |  |
|  | Min, Max | 0.00, 71.20 | 0.00, 58.00 |  |
| CD16^dim^;CD56^bright^ | N | 11 | 20 | 0.88 |
|  | Mean (s.d.) | 4.41 (9.15) | 2.77 (3.71) |  |
|  | Median (Q1, Q3) | 0.88 (0.00, 6.73) | 1.48 (0.00, 4.08) |  |
|  | Min, Max | 0.00, 30.90 | 0.00, 12.90 |  |
| CD16^dim^;CD56^dim^ | N | 11 | 20 | 0.55 |
|  | Mean (s.d.) | 11.68 (9.65) | 9.10 (7.89) |  |
|  | Median (Q1, Q3) | 8.31 (3.45, 21.30) | 8.11 (2.75, 12.45) |  |
|  | Min, Max | 0.22, 28.20 | 0.00, 26.50 |  |
| CD49A^+^;CD56^+^ | N | 11 | 20 | 0.40 |
|  | Mean (s.d.) | 29.73 (22.19) | 22.10 (17.60) |  |
|  | Median (Q1, Q3) | 30.30 (3.45, 50.60) | 18.75 (8.25, 31.95) |  |
|  | Min, Max | 0.33, 57.60 | 0.00, 58.70 |  |
| CD56+;NKG2A+ | N | 11 | 20 | 0.73 |
|  | Mean (s.d.) | 29.20 (25.85) | 24.27 (20.02) |  |
|  | Median (Q1, Q3) | 24.70 (0.45, 57.30) | 22.85 (5.18, 38.45) |  |
|  | Min, Max | 0.00, 66.30 | 0.00, 60.60 |  |
| CD56+;NKp46+ | N | 11 | 20 | 0.88 |
|  | Mean (s.d.) | 36.85 (31.94) | 32.83 (28.87) |  |
|  | Median (Q1, Q3) | 32.80 (0.11, 60.00) | 29.65 (5.09, 56.90) |  |
|  | Min, Max | 0.00, 91.60 | 0.00, 88.90 |  |
| CD56bright | N | 11 | 20 | 0.85 |
|  | Mean (s.d.) | 9.42 (16.54) | 6.77 (9.75) |  |
|  | Median (Q1, Q3) | 4.96 (0.00, 11.50) | 3.07 (0.00, 7.68) |  |
|  | Min, Max | 0.00, 56.80 | 0.00, 32.90 |  |
| CD56bright;CD57+ | N | 11 | 20 | 0.69 |
|  | Mean (s.d.) | 6.52 (10.09) | 5.80 (8.86) |  |
|  | Median (Q1, Q3) | 5.10 (0.00, 7.11) | 2.25 (0.00, 6.28) |  |
|  | Min, Max | 0.00, 34.50 | 0.00, 28.70 |  |
| CD56dim;CD57+ | N | 11 | 20 | 0.97 |
|  | Mean (s.d.) | 9.63 (11.64) | 7.23 (8.49) |  |
|  | Median (Q1, Q3) | 6.78 (0.00, 19.10) | 3.38 (0.60, 11.55) |  |
|  | Min, Max | 0.00, 35.50 | 0.00, 27.20 |  |
| CD56high;NKG2Ahigh | N | 11 | 20 | 0.98 |
|  | Mean (s.d.) | 9.95 (14.99) | 7.83 (10.46) |  |
|  | Median (Q1, Q3) | 7.16 (0.00, 15.40) | 4.22 (0.26, 9.48) |  |
|  | Min, Max | 0.00, 50.70 | 0.00, 35.10 |  |
| CD9+;CD56+ | N | 11 | 20 | 0.43 |
|  | Mean (s.d.) | 15.87 (13.07) | 10.83 (8.58) |  |
|  | Median (Q1, Q3) | 16.00 (0.33, 22.10) | 11.55 (2.82, 17.40) |  |
|  | Min, Max | 0.00, 43.70 | 0.00, 27.30 |  |
| *CD3-;CD20-;CD14-;CD56+* | | | | |
| CD57+ | N | 11 | 20 | 0.59 |
|  | Mean (s.d.) | 58.40 (26.47) | 56.30 (20.69) |  |
|  | Median (Q1, Q3) | 60.40 (48.60, 84.50) | 57.05 (44.40, 72.15) |  |
|  | Min, Max | 0.00, 92.30 | 0.00, 85.60 |  |
| NKG2A+ | N | 11 | 20 | 0.19 |
|  | Mean (s.d.) | 52.88 (18.66) | 62.12 (21.88) |  |
|  | Median (Q1, Q3) | 54.50 (37.20, 67.80) | 61.25 (51.85, 81.15) |  |
|  | Min, Max | 15.40, 78.20 | 5.36, 100.00 |  |
| CD16-;NKG2A+ | N | 11 | 20 | 0.27 |
|  | Mean (s.d.) | 15.97 (9.67) | 22.84 (16.64) |  |
|  | Median (Q1, Q3) | 16.40 (7.69, 22.70) | 18.45 (10.30, 34.30) |  |
|  | Min, Max | 0.00, 34.40 | 0.00, 56.30 |  |
| CD16+;NKG2A | N | 11 | 20 | 0.97 |
|  | Mean (s.d.) | 36.28 (18.59) | 38.02 (22.18) |  |
|  | Median (Q1, Q3) | 41.10 (19.00, 52.30) | 35.60 (23.30, 50.95) |  |
|  | Min, Max | 6.72, 61.50 | 2.98, 100.00 |  |
| CD16+;NKG2A- | N | 11 | 20 | 0.13 |
|  | Mean (s.d.) | 29.33 (16.27) | 21.45 (16.53) |  |
|  | Median (Q1, Q3) | 25.90 (23.30, 40.60) | 19.20 (10.45, 29.50) |  |
|  | Min, Max | 0.00, 57.10 | 0.00, 61.00 |  |
| CD49A-;NKG2A+ | N | 11 | 20 | 0.73 |
|  | Mean (s.d.) | 16.80 (18.59) | 17.47 (14.36) |  |
|  | Median (Q1, Q3) | 9.91 (2.45, 22.20) | 14.70 (6.06, 27.65) |  |
|  | Min, Max | 0.00, 53.00 | 0.00, 45.00 |  |
| CD49A+;NKG2A- | N | 11 | 20 | 0.04 |
|  | Mean (s.d.) | 26.88 (18.00) | 13.43 (10.60) |  |
|  | Median (Q1, Q3) | 26.20 (11.80, 38.10) | 11.70 (6.78, 18.25) |  |
|  | Min, Max | 3.10, 59.40 | 0.00, 45.60 |  |
| CD49A+;NKG2A+ | N | 11 | 20 | 0.40 |
|  | Mean (s.d.) | 36.03 (21.92) | 44.35 (21.70) |  |
|  | Median (Q1, Q3) | 36.30 (14.70, 57.50) | 44.80 (29.50, 62.45) |  |
|  | Min, Max | 0.00, 57.90 | 2.98, 79.70 |  |
| CD57high;NKG2Ahigh | N | 11 | 20 | 1.00 |
|  | Mean (s.d.) | 7.44 (7.76) | 6.63 (6.11) |  |
|  | Median (Q1, Q3) | 4.11 (2.50, 11.20) | 5.29 (1.38, 10.33) |  |
|  | Min, Max | 0.00, 24.20 | 0.00, 23.20 |  |
| CD9-;NKG2A+ | N | 11 | 20 | 0.48 |
|  | Mean (s.d.) | 41.49 (14.70) | 47.83 (20.01) |  |
|  | Median (Q1, Q3) | 42.50 (29.50, 52.10) | 44.20 (37.45, 61.75) |  |
|  | Min, Max | 11.50, 64.70 | 1.79, 100.00 |  |
| CD9+;NKG2A- | N | 11 | 20 | 0.31 |
|  | Mean (s.d.) | 15.30 (12.11) | 11.50 (12.76) |  |
|  | Median (Q1, Q3) | 8.86 (6.85, 30.60) | 8.35 (5.78, 12.05) |  |
|  | Min, Max | 0.00, 34.60 | 0.00, 57.70 |  |
| CD9+;NKG2A+ | N | 11 | 20 | 0.73 |
|  | Mean (s.d.) | 9.65 (8.36) | 11.54 (9.11) |  |
|  | Median (Q1, Q3) | 5.90 (3.85, 16.60) | 10.85 (3.70, 17.80) |  |
|  | Min, Max | 0.00, 26.30 | 0.00, 34.60 |  |
| *Not Applicable* | | | | |
| Lymphocytes | N | 11 | 18 | 0.75 |
|  | Mean (s.d.) | 67.47 (22.99) | 67.68 (23.59) |  |
|  | Median (Q1, Q3) | 74.90 (56.00, 79.30) | 76.90 (45.70, 84.30) |  |
|  | Min, Max | 6.73, 88.50 | 12.90, 96.50 |  |

*¹ Women who were on cART (3+ drugs) at conception or initiated cART at ≤ 3 weeks gestation AND there was no drug interruption during the whole 1st trimester AND there was no drug interruption within 6 weeks before specimen collection.*

*² Women who initiated cART at ≥ the 2nd trimester AND there was no ARV exposure at all at conception or the 1st trimester AND there was no drug interruption within 6 weeks before specimen collection.*

*³ Wilcoxon rank-sum test.*
